# When AI becomes a language partner: a moderated serial mediation model of AIGC-supported communicative task engagement and learning enjoyment

**DOI:** 10.3389/fpsyg.2026.1903870

**Published:** 2026-07-22

**Authors:** Jingwen Tang, Fei Wang

**Affiliations:** 1 Department of Early Childhood Education, Kangwon National University, Samcheok, Republic of Korea; 2 School of Journalism and Communication, China West Normal University, Nanchong, Sichuan, China

**Keywords:** AIGC, ambiguity tolerance, communicative task engagement, learning enjoyment, media richness, perceived epistemic value, social presence

## Abstract

**Background:**

Language learning is a deeply social and affective process, and much of it now unfolds beyond the classroom, through AI generated content (AIGC) tools that learners take up for communicative practice in everyday digital life. This rapid, largely unguided shift raises a timely question for the social and affective domain of language learning: whether practicing with an AI provides relational and emotional experiences that sustain language use, such as a sense of a responsive partner and the enjoyment of meaningful exchange, and how this process varies with learners’ tolerance of ambiguity.

**Methods:**

A correlational survey of 780 s or foreign language learners who use AIGC tools for communicative practice tested a moderated moderated serial mediation model linking communicative task engagement to learning enjoyment through social presence and then perceived epistemic value. Ambiguity tolerance served as a moderator, and the richness of the interaction channel (text, voice, or multimodal) as a further boundary condition.

**Results:**

Communicative task engagement was positively associated with learning enjoyment, and the results were consistent with an indirect association through social presence and then perceived epistemic value. The engagement associations were stronger for learners lower in ambiguity tolerance, a compensatory pattern that was more pronounced in the richer voice or multimodal subgroup, chiefly at lower and mean ambiguity tolerance. This channel pattern was statistically reliable for the associations with social presence and with perceived epistemic value. Because the study was cross-sectional, the findings should be interpreted with caution.

**Conclusion:**

Learning enjoyment in AIGC-supported communicative practice appears to be a social and affective achievement: it is stronger when engaged learners report a sense of a responsive partner and judge the activity worthwhile, and its association with engagement varies with their comfort with uncertainty and with the richness of the channel. By centering this experience, the study contributes to the social and affective domain of language learning beyond the classroom and locates the value of practicing with AI in the relational and emotional experience it affords. Tasks that invite responsive, conversational exchange and visible learning gains may help learners, especially those uneasy with uncertainty, sustain such practice.

## 1 Introduction

Opportunities to use a second or foreign language in genuine communication are often scarce. Class time is limited, contact with proficient interlocutors is uneven, and learners who fear making mistakes frequently hold back from speaking (Şahin Kızıl et al., 2025; Wiboolyasarin et al., 2025). AI-generated content (AIGC) tools, enabled by generative artificial intelligence and delivered through conversational systems such as ChatGPT, Kimi, Doubao, ERNIE Bot and other tools, have entered this gap at remarkable speed, offering input, feedback, and dialogue on demand and expanding the range of communicative practice available to learners beyond the people immediately at hand (McGrath et al., 2024; Mohebbi, 2025). Learners increasingly take up these tools on their own initiative, outside formal instruction, for role-play, dialogue practice, and rehearsal of language they expect to use elsewhere (Liu et al., 2024).

The speed of this uptake has outpaced understanding of its psychological value. More access to interaction is no guarantee of a better communicative experience. In fact, empirical evidence indicates that when generative systems misinterpret input, provide generic responses, or lack emotional resonance, the interaction can feel mechanical and isolating, which may frustrate learners and even heighten their communication anxiety (Annamalai et al., 2023; Çakmak, 2022; Wiboolyasarin et al., 2025). AIGC can multiply the quantity of practice, but whether—and under what conditions—that practice is experienced as socially responsive, epistemically worthwhile, and enjoyable remains an open question. Because practice of this kind is sustained largely by choice, the question of how it feels is not peripheral: it bears directly on whether learners continue to use these tools once their novelty has passed (Wu and Kabilan, 2025; Zhang and Liu, 2025).

Research on AIGC in language learning has grown quickly but has concentrated on the tool and its products. Reviews catalogue what AIGC can do across writing, speaking, vocabulary, and assessment, and the affordances of individualized input and repeated practice (Alhusaiyan, 2025; Chen et al., 2025; Lee et al., 2025; Li et al., 2025; Wang et al., 2025). What remains underdeveloped is an account of process: how a learner’s involvement with AIGC becomes, or fails to become, an experience worth returning to. Enjoyment is central to that question. In the positive-psychology turn in second language research, enjoyment is the most studied positive emotion and is associated with effort, persistence, and continued participation (Dewaele and Li, 2020; Wang et al., 2021; Wu and Kabilan, 2025; Zhang et al., 2024). Yet it has been examined largely as a classroom phenomenon, as a relatively static and individual trait, and seldom in settings where the interlocutor is a generative system or where practice is interactional and self-directed (Wu and Kabilan, 2025). Recent work on social grooming also suggests that everyday digital interaction can shape the affective side of language learning. Wang and Wang (2025a), for example, showed that foreign-language social grooming on social media was linked to learners’ enjoyment through social capital and social support, while related work on live photo-sharing showed that social grooming was associated with well-being through social capital and was conditioned by privacy need (Wang and Wang, 2025b).

*Three gaps follow*: First, research describes what AIGC does but not the process through which engagement with it relates to enjoyment, leaving little basis for designing practice that learners willingly sustain. Second, enjoyment has rarely been studied as an interactional outcome in learner-to-AI communication, so it is not known whether the felt experience of being in a meaningful exchange is what makes such practice enjoyable. Third, the conditions under which AIGC-supported communication is enjoyable remain unexamined, in particular how a learner difference in the tolerance of ambiguity and the richness of the interaction channel are associated with the strength of the process, so current guidance cannot identify who needs additional support or under which channel. Each gap corresponds to a practical need: sustaining voluntary practice, making solitary practice feel meaningful, and supporting learners least comfortable with uncertainty.

*Addressing these gaps is a theoretical task*: The control-value theory of achievement emotions holds that enjoyment is an activity emotion arising when a learner appraises an activity as both valuable and within their control (Pekrun, 2006); on this view enjoyment is a downstream result of appraisal, and a tool does not produce it directly. Situated expectancy-value theory adds that such value is judged in relation to the task and the moment (Eccles and Wigfield, 2020), positioning a learner’s judgment that an activity advances understanding—its perceived epistemic value (Peels, 2018)—as a proximal correlate of enjoyment. Because communication is relational, an interactional account is needed alongside: in mediated exchange, a sense of being with a responsive partner, namely social presence, shapes how the interaction is experienced (Kreijns et al., 2003; Makransky et al., 2017). Control-value theory and situated expectancy-value theory specify how a learner’s appraisals of value and control are associated with achievement emotions, yet they were developed for individual classroom achievement and address the relational quality of a mediated exchange only indirectly. Recent research emphasizes that relying solely on these individual-oriented appraisal frameworks risks neglecting the interpersonal and communicative nature of emotions (Wu and Kabilan, 2025; Zhang et al., 2024). Because communicative practice with an AIGC system is inherently interactional, an account of the relational experience is needed alongside these appraisal theories, and social presence supplies the crucial relational construct they leave unspecified. Together these ideas imply an ordering in which engagement is associated with social presence, which is associated with an appraisal of epistemic value, which is in turn associated with enjoyment. Finally, because appraisals of control are person-dependent, stable differences should condition the process, and the strength of that conditioning may itself depend on the richness of the channel through which communication takes place (Chu et al., 2015; McLain, 2009; Zhang and Zou, 2022).

This study therefore asks how, for whom, and through which channel AIGC-supported communicative task engagement is associated with learning enjoyment. It examines whether engagement relates to enjoyment serially, through social presence and then perceived epistemic value; whether tolerance of ambiguity conditions this indirect pathway, such that it is stronger for learners less tolerant of ambiguity; and whether this conditional pattern is more pronounced in richer, voice-based or multimodal interaction than in leaner, text-based exchange. In doing so, the study offers a process account of enjoyment in AIGC-supported communicative practice, treats enjoyment as an interactional achievement carried by social presence and perceived epistemic value, and specifies the learner-side and channel-side conditions under which the process is expected to be stronger.

## 2 Literature review and hypothesis development

The model is developed below as a sequence of conceptual links. Section 2.1 relates communicative task engagement to learning enjoyment. Sections 2.2 and 2.3 introduce social presence and perceived epistemic value as the relational and evaluative correlates through which that association is expected to operate, and Section 2.4 joins them into a sequence. Section 2.5 introduces tolerance of ambiguity as a person-level boundary condition, and Section 2.6 introduces interaction mode as a channel-level boundary condition grounded in media richness and multimodality.

### 2.1 AIGC-supported communicative task engagement and learning enjoyment

Engagement is widely regarded as a central construct in second language learning, distinct from mere participation and understood as a multidimensional state encompassing behavioral, cognitive, emotional, agentic, and social involvement in a task (Hiver et al., 2024; Mercer and Dörnyei, 2020; Philp and Duchesne, 2016; Reeve and Tseng, 2011). In task-based work specifically, communicative task engagement refers to the quality of a learner’s involvement while carrying out a communicative task, and it has been linked to the design and conduct of the task as well as to learner characteristics (Egbert et al., 2021; Qiu, 2025; Zare and Derakhshan, 2025). Foreign language enjoyment, in turn, is the positive activity emotion learners feel when an activity is absorbing, meaningful, and within reach, and it has been associated with sustained effort and willingness to continue (Dewaele and MacIntyre, 2014; Dewaele and Li, 2020; Wang et al., 2021).

The control-value theory of achievement emotions provides a reason to expect these two constructs to be related. In that account, activity emotions such as enjoyment arise when learners are absorbed in an activity they appraise as controllable and valuable (Pekrun, 2006). Engagement, as sustained and self-directed involvement in a communicative task, is the experiential condition under which such appraisals are most likely to be made, so deeper engagement is expected to accompany greater enjoyment. Empirically, enjoyment and engagement co-occur across classroom and technology-supported settings, including engagement in AI-mediated learning examined through conditional process models (Cai and Xing, 2025; Fan and Zhang, 2024; Lei et al., 2026; Yang et al., 2026; Zhang, 2024; Zhang et al., 2024), and in AIGC-supported communicative practice—where involvement is voluntary and self-paced—engagement is treated here as a proximal learner-side correlate of how the activity is experienced. This study therefore treats AIGC-supported communicative task engagement as the focal antecedent of learning enjoyment.

*H1*: AIGC-supported communicative task engagement is positively associated with learning enjoyment (total association).

### 2.2 Social presence as an interactional correlate

If engagement is associated with enjoyment, the question becomes which relational qualities of engaged communicative practice are linked to that enjoyment. Because communication is inherently relational, one answer lies in the relational quality of the exchange. Social presence refers to the sense of being together with a real, responsive partner in mediated communication—the feeling that there is someone there, not merely a mechanism being operated (Kreijns et al., 2003; Makransky et al., 2017). In computer-supported settings, the absence of socio-emotional cues has long been identified as a reason that otherwise well-designed environments fail to engage learners, whereas a sense of social presence is associated with more positive experience and continued involvement (Kreijns et al., 2003).

*Two links follow*: First, social presence is not a fixed property of a tool but something constructed through a learner’s own sustained, responsive interaction; more engaged communicative practice—turn-taking, meaning negotiation, feedback-seeking—affords more of the cues from which a sense of a present partner is built (Makransky et al., 2017). In AIGC-supported communication, where the system produces fluent, contingent, conversational responses, engaged interaction is expected to be accompanied by a stronger sense of social presence. Second, this relational experience is associated with how the activity feels: a sense of communicating with a responsive partner has been linked to more positive learning experience in chatbot-assisted English learning (Hong et al., 2024). How learners respond to an artificial as opposed to a human communicative partner has likewise been shown to depend on their motivation, the interaction context, and the particular conversational AI system used (Lin et al., 2026; Wang et al., 2024). Social presence is therefore proposed as a relational correlate situated between engagement and enjoyment. Conceptually, social presence is distinct from the social-interactive dimension of engagement: the latter comprises observable communicative behaviors during the task, such as turn-taking and feedback-seeking (Hiver et al., 2024; Qiu, 2025; Zare and Derakhshan, 2025), whereas social presence is the subjective perception that a responsive partner is present (Hong et al., 2024; Makransky et al., 2017).

*H2*: Social presence functions as an intervening variable in the association between AIGC-supported communicative task engagement and learning enjoyment.

### 2.3 Perceived epistemic value as an evaluative correlate

A relational experience alone does not capture why communicative practice is judged worthwhile. Control-value theory specifies that enjoyment depends not only on involvement but on the value a learner attaches to the activity (Pekrun, 2006), and situated expectancy-value theory locates such value in the learner’s appraisal of the specific task at hand (Eccles and Wigfield, 2020). Perceived epistemic value names the appraisal most relevant to language practice: a learner’s judgment that an activity has advanced their knowledge, understanding, and insight—here, that AIGC-supported communicative tasks have helped them gain language knowledge and understand how the target language is used (Hong et al., 2016; Peels, 2018; Tantucci et al., 2022). This appraisal is conceptually distinct from cognitive engagement, which is an in-task process of effortful processing, and from hedonic value, which is the pleasure of the activity itself (Hong et al., 2016; Philp and Duchesne, 2016). Two links again follow. Engaged communicative practice, in which a learner actively produces language, seeks feedback, and works through meaning, provides the experiential basis for judging that the activity has yielded understanding, so engagement is expected to be associated with stronger perceived epistemic value. That appraisal, in turn, is a proximal correlate of enjoyment: activities judged to advance understanding are experienced as worthwhile and absorbing (Eccles and Wigfield, 2020; Hong et al., 2024; Pekrun, 2006). Perceived epistemic value is therefore proposed as an evaluative correlate situated between engagement and enjoyment.

*H3*: Perceived epistemic value functions as an intervening variable in the association between AIGC-supported communicative task engagement and learning enjoyment.

### 2.4 The sequential pathway from engagement to enjoyment

Social presence and perceived epistemic value are not independent. Work on experiential and perceived value suggests an ordering in which a relational, socio-emotional experience is associated with, and may set the stage for, an evaluative appraisal: when learners feel they are in a responsive exchange, they are better positioned to judge that the exchange has advanced their understanding (Hong et al., 2016, 2024; Kreijns et al., 2003). The socio-emotional-first logic is also consistent with accounts in which attention to social interaction is a precondition for the cognitive work of a learning environment to register as valuable (Kreijns et al., 2003). Reading these together with control-value theory implies a sequence: engagement is associated with social presence, social presence is associated with perceived epistemic value, and perceived epistemic value is associated with enjoyment. The relational experience and the evaluative appraisal thus form an ordered pathway linking engaged practice to how it is enjoyed (Hong et al., 2024).

*H4*: AIGC-supported communicative task engagement is associated with learning enjoyment through a sequential pathway in which social presence is associated with perceived epistemic value.

### 2.5 Ambiguity tolerance as a person-level boundary condition

Because appraisals of value and control are made by particular learners, the process above should not hold uniformly. Tolerance of ambiguity—the capacity to handle uncertain, incomplete, or inconsistent information without undue discomfort—is a stable individual difference of particular relevance where uncertainty is intrinsic to the activity (Chu et al., 2015; McLain, 2009). AIGC-supported communication is such an activity: generated output is variable and at times inaccurate, so interacting with it requires working through uncertainty (Wiboolyasarin et al., 2025). The role of ambiguity tolerance here is best understood as compensatory. For learners more tolerant of ambiguity, uncertain exchanges are manageable with relatively little sustained effort, so their experience depends less on how engaged they are. For learners less tolerant of ambiguity, the same uncertainty may be more disruptive, and active engagement may be more strongly associated with a sense that the exchange remains manageable; for these learners, engagement is expected to matter most for sustaining a sense of social presence, an appraisal of epistemic value, and enjoyment (Aslan and Thompson, 2021; Chen, 2023; Zhang, 2024). On this reading, ambiguity tolerance negatively moderates the engagement-related associations: they are stronger at lower levels of ambiguity tolerance. This is consistent with evidence that ambiguity tolerance negatively moderated the association between engagement and learning effectiveness in technology-supported language learning (Zhang, 2024). This person-by-situation logic is theoretically anchored in cognitive load theory (Sweller, 1988), which posits that the value and effectiveness of additional support depend heavily on the learner’s prior capacity to manage task-induced demands (Zhang and Zou, 2022). In highly uncertain environments, responsive AIGC interaction may function as a potential compensatory resource for learners who tolerate ambiguity less easily (Yu et al., 2022), in the same way that external scaffolding can be more relevant when learners lack internal resources for managing task demands (Lei et al., 2026).

*H5*: Ambiguity tolerance negatively moderates the association between AIGC-supported communicative task engagement and social presence (5a), perceived epistemic value (5b) and learning enjoyment (5c), such that the association is stronger at lower levels of ambiguity tolerance.

Because social presence and perceived epistemic value are expected to convey part, but not all, of the association between engagement and enjoyment, a residual direct association remains, and ambiguity tolerance is expected to condition this residual association as well. For learners with lower ambiguity tolerance, uncertain learning situations are often viewed as immediate sources of psychological discomfort (Chu et al., 2015). In such ambiguous environments, active task engagement may serve as a compensatory and self-regulatory resource and may remain associated with enjoyment even after relational and evaluative appraisals are considered (Yu et al., 2022; Zhang, 2024).

If ambiguity tolerance conditions the first link in the sequence, that conditioning should also be carried along the sequence as a whole. When engagement is more strongly associated with social presence for less tolerant learners, the downstream associations through perceived epistemic value to enjoyment should likewise be more pronounced for them, so that the serial indirect association is conditional on ambiguity tolerance (Pekrun, 2006; Zhang, 2024).

*H6*: The serial indirect association of AIGC-supported communicative task engagement with learning enjoyment, through social presence and then perceived epistemic value, is moderated by ambiguity tolerance, such that it is stronger at lower levels of ambiguity tolerance.

### 2.6 Interaction mode as a boundary condition: multimodality and media richness

The strength of this compensatory pattern may itself depend on the channel through which AIGC-supported communication takes place. Such tasks can be carried out through text, voice, or multimodal exchange, and modes of multimedia input have been shown to differ in their effectiveness for second language learning (Zhang and Zou, 2022). Voice and multimodal exchange are richer in the sense that they carry more communicative cues and afford greater immediacy: voice conveys prosody, timing, and turn-taking signals, and multimodal exchange adds visual cues, supplying more of the information from which a sense of a present partner is built (Daft and Lengel, 1986). Such richer input has been associated with deeper processing, heightened engagement, and a more positive learning experience, and responsive AIGC interaction has been linked to a stronger sense of social presence (Hong et al., 2024; Wang et al., 2026; Zhang and Zou, 2022). Greater richness is not uniformly advantageous, however. Drawing on cognitive load theory (Sweller, 1988), the same immediacy and multiplicity of cues raise processing demands, since real-time speech and multimodal input are less easily paused or re-read (Wang et al., 2026; Zhang and Zou, 2022), so the net contribution is expected to depend on the learner’s capacity to tolerate ambiguity. For learners less comfortable with ambiguity, this may be where engagement-related associations are strongest, because active engagement may be linked to a more manageable exchange that would otherwise feel uncertain (Zhang, 2024). In leaner, text-based interaction, uncertainty is more easily managed without sustained engagement, and the contribution of engagement may be weaker for these learners. Because the field has also called for closer attention to how learner factors interact with features of multimedia input (Zhang and Zou, 2022), the compensatory associations proposed for lower-tolerance learners may be more pronounced in richer channels. The following hypothesis is proposed.

*H7*: The negative moderation of ambiguity tolerance on the associations between AIGC-supported communicative task engagement and social presence, perceived epistemic value, and learning enjoyment is stronger in richer, voice-based or multimodal interaction than in leaner, text-based interaction.

Because the serial sequence carries this first relational link forward through appraisal to emotion, a channel-related difference at the link may extend across the sequence. Richer, multimodal input has been associated with deeper processing and stronger engagement (Wang et al., 2026; Zhang and Zou, 2022), so if engagement is more strongly associated with social presence under richer channels for learners with lower ambiguity tolerance, that advantage may be transmitted through perceived epistemic value to enjoyment (Hong et al., 2024; Zhang, 2024). In leaner, text-based interaction, where uncertainty can be resolved with less sustained engagement, the conditional indirect association may be correspondingly weaker. The following hypothesis is proposed.

*H8*: The conditional indirect association of AIGC-supported communicative task engagement with learning enjoyment, through social presence and then perceived epistemic value, is more pronounced in richer, voice-based or multimodal interaction than in leaner, text-based interaction, and remains stronger at lower levels of ambiguity tolerance.

**Figure 1:**
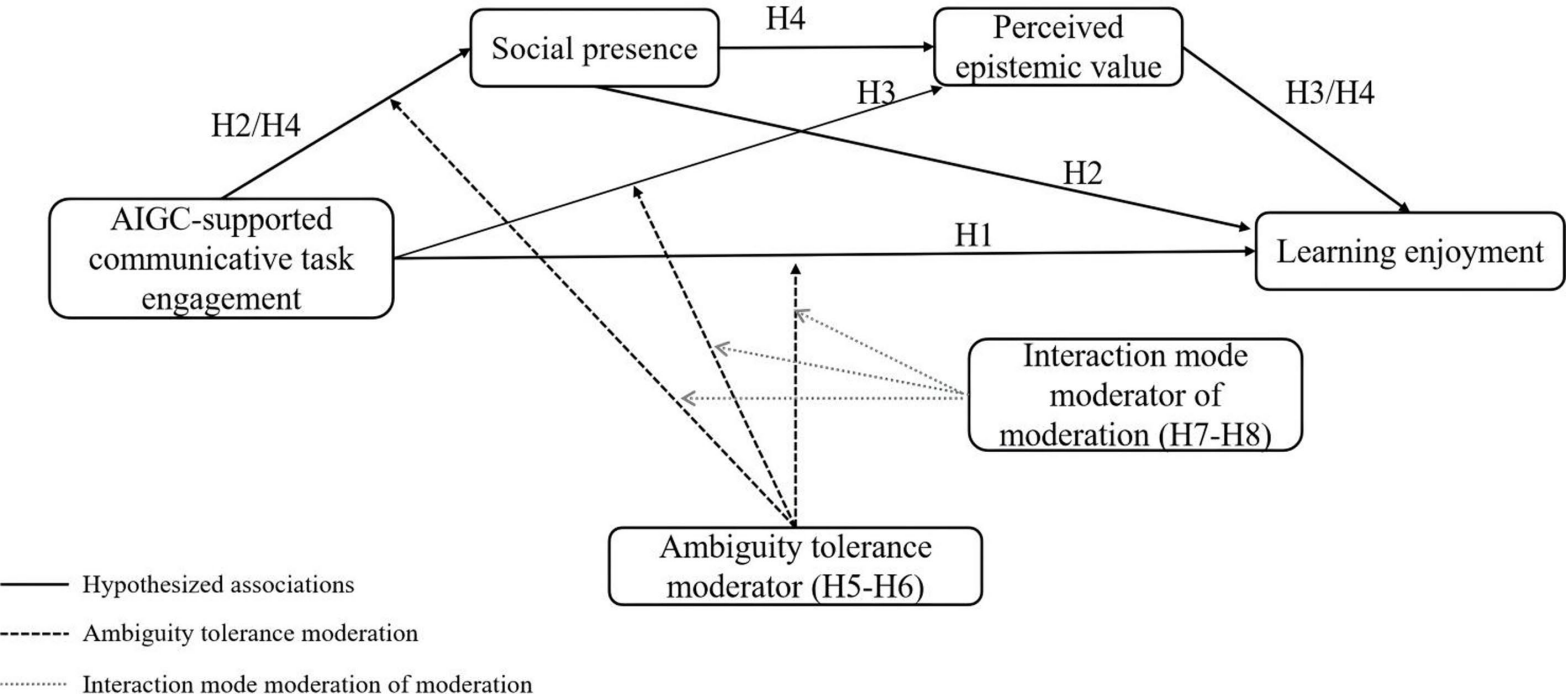
Theoretical pathway. Solid arrows represent the hypothesized associations among the focal constructs: communicative task engagement, social presence, perceived epistemic value, and learning enjoyment. The dark dashed arrows indicate moderation by ambiguity tolerance of the engagement associations with social presence, perceived epistemic value, and learning enjoyment, as well as the corresponding conditional serial indirect association (H5–H6). The light dotted arrows indicate the further moderation by interaction mode—text-based only versus richer voice-based or multimodal interaction—tested as the three-way interaction terms and the between-group difference in the conditional serial indirect association (H7–H8). Ambiguity tolerance and interaction mode are moderators rather than variables in the serial chain.

Figure 1 shows the theoretical pathway of this study.

## 3 Methods

### 3.1 Participants and procedure

This study adopted a quantitative, cross-sectional questionnaire design to examine the associations among AIGC-supported communicative task engagement, social presence, perceived epistemic value, learning enjoyment, and ambiguity tolerance in second/foreign language learning. Before data collection, ethical approval was obtained from the Academic Ethics Committee of the School of Journalism and Communication, China West Normal University (Approval No. XHSDJC202604001). All participants provided informed consent before completing the questionnaire. Participation was voluntary, and participants were informed that they could withdraw at any time. No personally identifying information was collected.

Participants were recruited and the data were collected by Chongqing Rounen Film and Television Media Co., Ltd. (Chongqing, China), a professional media market research company. Data collection was conducted through the company’s online survey platform under quality-control requirements specified by the researchers. These controls included permitting only one response per device, an embedded attention-check item, and a minimum completion-time filter of 5 minutes. The company was responsible for participant recruitment, questionnaire distribution, response monitoring, and initial data export. The researchers designed the questionnaire, specified eligibility criteria, and conducted all subsequent data screening and statistical analyses.

A total of 825 responses were collected. Attentiveness was screened with a single instructed-response item embedded among the focal items and answered on the same five-point scale; participants were instructed to select a specified option, and the 25 responses (3.0%) that selected a different option were removed. Among attention-check-valid responses, 20 cases gave the same response option to every one of the 39 focal items (zero within-person variance across focal items) and were removed as invariant responders. The final analytic sample consisted of 780 valid responses. Sparse focal-item missingness was observed, with 224 missing cells across all focal scale items, accounting for 0.74% of the focal-item data. Given the low missing rate, item-level missing values were replaced with the corresponding item median before scale construction.

The questionnaire was developed bilingually in English and Chinese. The initial English version was translated into Chinese by a bilingual researcher familiar with applied linguistics and educational psychology. A second bilingual researcher then back-translated the Chinese version into English. The original English version and the back-translated version were compared, and discrepancies were discussed and resolved to ensure semantic equivalence, conceptual clarity, and appropriateness for the AIGC-supported communicative language-learning context.

The analytic sample (*N* = 780) comprised adult second or foreign language learners recruited in mainland China; all were aged 18 or above (18–22 years, 60.0%; 23–26, 29.1%; 27 or above, 10.9%). First language was not recorded in the questionnaire; however, participants were recruited in mainland China and completed a Chinese–English bilingual questionnaire. In terms of gender, 412 participants were female (52.8%), 322 were male (41.3%), and 46 preferred not to report (5.9%). Most participants were undergraduates (60.8%), followed by master’s-level postgraduates (28.3%), those with high school or below (6.0%), and doctoral students (4.9%). English was the most common target language (57.9%), followed by Japanese (16.0%), European languages (11.3%), Korean (10.5%), and other languages (4.2%). Self-rated proficiency was most often intermediate (63.1%), with fewer participants reporting advanced (19.1%) or beginner (17.8%) proficiency. The most frequently used primary AIGC tool was ChatGPT (38.7%), followed by Kimi (19.5%), ERNIE Bot (16.0%), Doubao (15.3%), and other tools (10.5%). For interaction mode, 271 participants (34.7%) reported text-based AIGC interaction only, whereas 509 (65.3%) reported richer voice-based or multimodal interaction; the richer-interaction group combined those who reported text + voice and those who reported text + voice + visual/video or other multimodal cues. A full demographic and background profile of the sample is provided in Supplementary Appendix B.

### 3.2 Measurements

Unless otherwise specified, focal items were rated on a five-point Likert scale from 1 (strongly disagree) to 5 (strongly agree), and item responses were averaged to form composite scores, with higher scores indicating higher levels of the construct. All items were contextualized to AIGC-supported communicative tasks, defined as second or foreign language learning activities in which learners use generative AI tools for communicative practice, such as role-play, dialogue practice, meaning negotiation, preparation for real communication, or feedback-seeking on language use. To make explicit which construct each set of items measures, the item-to-construct mapping is stated for every measure below.

AIGC-supported communicative task engagement (the focal independent variable) was measured with 16 items adapted from the Task Engagement Questionnaire (TEQ; Zare and Derakhshan, 2025), whose development and validation identified a five-dimension structure—emotional, agentic, behavioral, social, and cognitive engagement (25 validated items). Four of these dimensions were used and were modeled as a single higher-order construct: behavioral engagement (items B1–B4), cognitive engagement (items B5–B8), agentic engagement (items B9–B12), and social-interactive engagement (items B13–B16; the social engagement dimension of the TEQ). Behavioral engagement concerns effort, attention, and persistence; cognitive engagement concerns deliberate, strategic processing; agentic engagement concerns a learner’s constructive, proactive contribution to the interaction (Reeve and Tseng, 2011); and social-interactive engagement concerns communicative, interpersonal involvement in the exchange. The emotional engagement dimension of the TEQ was not used, because emotional experience is represented by the outcome variable, learning enjoyment. The four retained dimensions formed a coherent higher-order engagement construct that fit the data well (reported below), so omitting the emotional dimension appeared not to compromise the integrity of the construct in the present data. Sample items include “I stay focused and avoid distraction during AIGC-supported communicative tasks” (behavioral) and “During AIGC-supported communicative tasks, I ask the AI for feedback on my language use” (agentic).

Social presence (first intervening variable) was measured with four items (C1–C4) adapted from the social-presence dimension of the Multimodal Presence Scale (Makransky et al., 2017), capturing the perception that the AIGC system functioned as a socially responsive communicative partner. A sample item is “When I interact with the AIGC system, I feel as if another communicative partner is present.”

Perceived epistemic value (second intervening variable) was measured with five items (D1–D5) adapted from existing operationalizations of epistemic and perceived value (Hong et al., 2016, 2024) and grounded in Peels (2018) conceptualization of epistemic value, contextualized to AIGC-supported communicative tasks. The items assess perceived gains in language knowledge, understanding of communicative language use, and insight into meaning expression. During the translation and back-translation procedure, bilingual researchers in applied linguistics reviewed the items for semantic equivalence, conceptual clarity, and appropriateness for the AIGC-supported communicative language-learning context. A sample item is “AIGC-supported communicative tasks have helped me understand how the target language is used in real communication.” Because the items were assembled and adapted for a new context, their structure was further examined through the exploratory and confirmatory factor analyses reported below.

Learning enjoyment (the dependent variable) was measured with six items (E1–E6) adapted from the Foreign Language Learning Enjoyment Scale (Aydın et al., 2024), capturing enjoyment, interest, and overall positive experience during AIGC-supported communicative learning. A sample item is “Overall, I find AIGC-supported communicative tasks enjoyable.”

Ambiguity tolerance (the moderator) was measured with eight items (F1–F8) adapted from second-language ambiguity-tolerance research, in particular Chu et al.’s (2015) use of Ely’s Second Language Tolerance of Ambiguity Scale (Ely, 1995). Items were contextualized to AIGC-supported communication and worded so that higher scores indicate greater tolerance of ambiguity. Specifically, each item was reworded to refer to uncertainty arising during interaction with an AIGC system, rather than to ambiguity in second-language learning in general. A sample item is “It does not bother me when an AI response in the target language is not completely clear.” Covariates were gender, self-rated target-language proficiency (1 = beginner, 2 = intermediate, 3 = advanced), and frequency of AIGC use for communicative tasks, each treated as a categorical variable. Interaction mode was recoded into two groups, leaner text-based interaction only and richer voice-based or multimodal interaction, for the heterogeneity analyses; construct abbreviations and the coding of all variables are summarized in Supplementary Appendix A.

### 3.3 Data analysis

All analyses were conducted in Python. After screening, responses failing the attention check or showing invariant focal responding were removed; sparse item-level missing values on focal items (0.74% of focal-item responses) were replaced with the item median before composites were computed. The choice of single imputation is defensible given the very low missingness, although full-information or multiple imputation would be preferable in datasets with more missing data.

The measurement model was examined with a split-sample procedure: the analytic sample was randomly divided into an exploratory factor analysis subsample and a confirmatory factor analysis subsample. Exploratory factor analysis used maximum-likelihood extraction with promax rotation. Confirmatory factor analysis specified behavioral, cognitive, agentic, and social-interactive engagement as first-order factors loading on a higher-order task-engagement factor, with social presence, perceived epistemic value, learning enjoyment, and ambiguity tolerance as separate factors. Internal consistency was assessed with Cronbach’s alpha; convergent validity with standardized loadings, composite reliability, and average variance extracted; and discriminant validity with the Fornell–Larcker criterion and the heterotrait–monotrait ratio. Common method bias was examined with Harman’s single-factor diagnostic and a single-factor confirmatory model; these are diagnostics only, and common method bias cannot be ruled out under a cross-sectional self-report design.

The moderated serial mediation model was estimated with ordinary least squares regression using heteroscedasticity-consistent (HC3) standard errors; this conditional process approach follows recent applications in AI-mediated learning and technology-adoption research (Lei et al., 2026; Lin et al., 2026). Task engagement was the focal independent variable, social presence the first intervening variable, perceived epistemic value the second, learning enjoyment the dependent variable, and ambiguity tolerance the moderator of the associations from engagement to social presence, perceived epistemic value, and enjoyment. Continuous predictors were mean-centered before forming product terms. Indirect, conditional indirect, and serial indirect associations, and the index of moderated serial mediation, were estimated with 5,000 bootstrap resamples using percentile confidence intervals; conditional associations were evaluated at the mean and at one standard deviation below and above the mean of ambiguity tolerance.

Interaction-mode heterogeneity was examined in three steps. First, as a preliminary measurement-comparability check, the hypothesized measurement model was fitted separately within the text-based and richer-interaction groups. Second, H7 was tested with full-sample regressions including the three-way product of task engagement, ambiguity tolerance, and interaction mode in the social-presence, perceived-epistemic-value, and enjoyment equations. Third, H8 was tested by computing the index of moderated serial mediation separately within each interaction-mode group and bootstrapping the difference between the two group-specific indices. Because interaction mode was self-reported and not experimentally assigned, these analyses are interpreted as subgroup heterogeneity analyses rather than as evidence of modality effects.

The raw interaction-mode item contained three response categories: text-based only, text + voice, and text + voice + visual/video or other multimodal cues. For the H7–H8 analyses, the latter two categories were combined into a richer-interaction group because both involved non-textual communicative cues and were theoretically aligned with the contrast between leaner text-based interaction and richer interaction. Thus, interaction mode was analyzed as a binary grouping variable: 0 = text-based only and 1 = richer interaction. The raw three-category distribution is reported in Supplementary Appendix B.

## 4 Results

### 4.1 Data screening and descriptive statistics

Of 825 submitted responses, 25 were removed for failing the attention check and 20 for showing invariant answers across all focal scale items, leaving a final analytic sample of 780. Item-level missingness on focal variables was 0.74% and was replaced with the item median before composite scores were computed. Descriptive statistics for the five focal variables appear in Table 1, where the means are reported without assigning qualitative labels such as moderate or high. Skewness ranged from −0.58 to −0.16 and kurtosis from −0.61 to −0.15, within the bounds conventionally regarded as acceptable for maximum-likelihood and regression-based estimation. Item-level descriptive statistics for all 39 items, including corrected item–total correlations and rotated factor loadings, are reported in Supplementary Appendix C, and the demographic profile of the sample is reported in Supplementary Appendix B.

**Table 1:** Descriptive statistics for the focal variables (*N* = 780).

Variable	M	SD	Min	Max	Skewness	Kurtosis
Communicative task engagement	3.42	0.70	1.19	5.00	−0.29	−0.32
Social presence	3.24	0.88	1.00	5.00	−0.16	−0.51
Perceived epistemic value	3.41	0.87	1.00	5.00	−0.32	−0.61
Learning enjoyment	3.59	0.88	1.00	5.00	−0.58	−0.15
Ambiguity tolerance	3.47	0.87	1.00	5.00	−0.35	−0.57

### 4.2 Measurement validation

The measurement model was examined through a split-sample validation procedure. To reduce the risk of capitalizing on sample-specific covariance patterns, the analytic sample was randomly divided into an exploratory subsample (*n* = 390) and an independent confirmatory subsample (*n* = 390). The measurement evaluation included sampling adequacy, exploratory factor analysis, confirmatory factor analysis, internal-consistency reliability, convergent validity, discriminant validity, common method diagnostics, and a preliminary measurement-comparability check across interaction modes.

Sampling adequacy and exploratory structure. The data were well suited to factor analysis: the Kaiser–Meyer–Olkin measure was 0.951, and Bartlett’s test of sphericity was significant, χ^2^(741) = 8928.21, *p* < 0.001. Maximum-likelihood extraction with promax rotation was used. The first eight eigenvalues were 13.82, 3.79, 2.42, 1.66, 1.38, 1.29, 1.16, and 1.00. An eight-factor solution was retained because it corresponded to the hypothesized measurement structure: four engagement dimensions, social presence, perceived epistemic value, learning enjoyment, and ambiguity tolerance. The eight factors accounted for 56.86% of the total variance. Because the eighth eigenvalue stood at the conventional unit threshold, factor retention did not rest on the eigenvalue criterion alone. A seven-factor solution was also inspected. It differed from the eight-factor solution mainly in that perceived epistemic value and learning enjoyment loaded together on a single factor, while the other constructs remained distinct. The seven-factor solution was not retained because perceived epistemic value and learning enjoyment are theoretically separable and were empirically discriminable in the confirmatory analyses. In the CFA, the hypothesized model with separate perceived-epistemic-value and learning-enjoyment factors fit the data well; in the discriminant-validity analysis, the Fornell–Larcker criterion was satisfied and the HTMT ratio between perceived epistemic value and learning enjoyment remained below 0.85. The eight-factor solution was therefore retained because it reproduced the hypothesized measurement design and showed a cleaner simple-loading pattern. Every item loaded on its intended factor, with primary loadings ranging from 0.55 to 0.88, and no item showed a secondary loading of 0.30 or above on any other factor. Because no secondary loading reached 0.30, none approached the more stringent 0.40 or 0.50 thresholds either. The rotated pattern therefore supported a clean simple structure, including empirical separation between perceived epistemic value and cognitive engagement. The full rotated pattern matrix is reported in Supplementary Appendix C.

Confirmatory factor analysis. In the independent confirmatory subsample, the higher-order measurement model fit the data well, χ^2^(688) = 761.49, χ^2^/df = 1.11, CFI = 0.990, TLI = 0.989, RMSEA = 0.017. In this model, behavioral, cognitive, agentic, and social-interactive engagement loaded on a higher-order communicative task engagement factor, while social presence, perceived epistemic value, learning enjoyment, and ambiguity tolerance were specified as distinct factors. These results supported retaining communicative task engagement as one higher-order construct measured by four first-order dimensions.

Reliability and convergent validity. Table 2 reports reliability and convergent-validity statistics for the focal constructs used in the structural analyses. Because communicative task engagement was modeled as a higher-order construct, the CTE row reports the reliability and validity statistics for the higher-order construct, with the four first-order engagement dimensions serving as its indicators. Detailed results for behavioral, cognitive, agentic, and social-interactive engagement are reported in Supplementary Appendix D. Cronbach’s alpha values ranged from 0.78 to 0.91, and composite reliability values ranged from 0.82 to 0.91, exceeding the conventional 0.70 criterion. AVE values ranged from 0.52 to 0.57 for the focal constructs reported in the main text, supporting convergent validity. Supplementary Appendix D further shows that the first-order engagement dimensions also showed acceptable reliability. Although the AVE for agentic engagement was slightly below 0.50, its composite reliability exceeded 0.70 and its standardized loadings were acceptable; therefore, the dimension was retained.

**Table 2:** Reliability and convergent validity.

Construct	Items	α	CR	AVE	Loading range
Communicative task engagement	4 dimensions / 16 items	0.78	0.82	0.54	0.68–0.77
Social presence	4	0.84	0.82	0.54	0.69–0.81
Perceived epistemic value	5	0.86	0.84	0.52	0.56–0.81
Learning enjoyment	6	0.89	0.87	0.53	0.63–0.83
Ambiguity tolerance	8	0.91	0.91	0.57	0.70–0.81

α, Cronbach’s alpha; CR, composite reliability; AVE, average variance extracted. For communicative task engagement, the reported values refer to the higher-order construct, with behavioral, cognitive, agentic, and social-interactive engagement as first-order indicators. Detailed first-order results are reported in Supplementary Appendix D.

Discriminant validity. Discriminant validity was assessed with the Fornell–Larcker criterion and the heterotrait–monotrait ratio of correlations (HTMT; Table 3). For every focal construct, the square root of the AVE exceeded that construct’s correlations with the others, and all HTMT ratios fell below the conservative 0.85 criterion. The highest ratio, between perceived epistemic value and learning enjoyment (HTMT = 0.81; *r* = 0.70), reflects the close theoretical relation modeled between an evaluative appraisal and the emotion it is expected to precede; it remained within accepted bounds, and the two were retained as distinct constructs. Conceptually, the two constructs remain distinct in kind, an evaluative appraisal of knowledge gain versus the positive emotion that appraisal is expected to precede; the strong association is consistent with this ordering rather than indicating redundancy, and the same pair was the only one to merge under a forced seven-factor solution.

**Table 3:** Discriminant validity: correlations, square root of AVE (diagonal), and HTMT (in parentheses).

Variables	CTE	SP	PEV	LE	AT
CTE	0.73	(0.60)	(0.65)	(0.67)	(0.33)
SP	0.52	0.73	(0.68)	(0.71)	(0.35)
PEV	0.57	0.58	0.72	(0.81)	(0.43)
LE	0.60	0.61	0.70	0.73	(0.41)
AT	0.29	0.30	0.38	0.37	0.76

CTE, communicative task engagement; SP, social presence; PEV, perceived epistemic value; LE, learning enjoyment; AT, ambiguity tolerance. Below-diagonal values are correlations; diagonal values are the square root of AVE; parenthetical values above the diagonal are HTMT ratios.

Common method bias. Two diagnostics were examined. Harman’s single-factor test indicated that the first unrotated factor accounted for 32.78% of the variance, below the 50% threshold. A one-factor confirmatory model also fit the data poorly, χ^2^(702) = 7411.41, CFI = 0.581, TLI = 0.558, RMSEA = 0.111, far worse than the hypothesized measurement model. These diagnostics did not indicate a dominant common method factor. Nevertheless, as in any cross-sectional self-report study, common method bias cannot be excluded entirely, and a marker-variable approach is recommended for future replication.

Measurement comparability across interaction modes. Because Hypotheses 7 and 8 compare patterns across interaction modes, measurement comparability was examined before the subgroup analyses. The configural model fit well in both groups: text-based group, *n* = 271, χ^2^(674) = 737.80, CFI = 0.988, TLI = 0.987, RMSEA = 0.019; richer-interaction group, *n* = 509, χ^2^(674) = 793.05, CFI = 0.989, TLI = 0.988, RMSEA = 0.019 (Supplementary Appendix E). These results indicate that the same broad factor structure was tenable across the two interaction-mode groups. Because full metric and scalar invariance were not established in the present analysis, the interaction-mode comparisons are interpreted as exploratory subgroup comparisons rather than as strong tests of measurement-equivalent group differences.

### 4.3 Direct association

With learning enjoyment regressed on communicative task engagement and the covariates (gender, self-rated proficiency, and frequency of AIGC use for communicative tasks, all treated as categorical), engagement was positively associated with enjoyment, *b* = 0.76, SE = 0.04, *p* < 0.001, 95% CI [0.69, 0.84], and the model accounted for 37.0% of the variance in enjoyment. H1 was supported. This total association declined to a direct association of *b* = 0.31 once social presence and perceived epistemic value were entered (Table 4), indicating that much of it operated through the intervening variables rather than reflecting a uniform inflation of associations; together with the common-method diagnostics reported above, this makes a dominant common-method explanation less likely, although common method bias cannot be ruled out under the present design.

**Table 4:** Regression coefficients for the three structural equations.

Predictor	→ Social presence	→ Perceived epistemic value	→ Learning enjoyment
Engagement (CTE)	0.61 (0.04)^***^	0.44 (0.04)^***^	0.31 (0.04)^***^
Ambiguity tolerance (AT)	0.15 (0.03)^***^	0.17 (0.03)^***^	0.08 (0.03)^**^
Engagement × AT	−0.22 (0.04)^***^	−0.13 (0.04)^**^	−0.17 (0.03)^***^
Social presence	—	0.32 (0.03)^***^	0.21 (0.03)^***^
Perceived epistemic value	—	—	0.39 (0.03)^***^
*R* ^2^	0.33	0.47	0.61

Unstandardized coefficients with HC3 standard errors in parentheses. Engagement and ambiguity tolerance were mean-centered. Covariates (gender, proficiency, AIGC-use frequency) were included in every equation; full covariate coefficients are reported in Supplementary Appendix F. The direct-association model (H1: enjoyment on engagement only) yielded *b* = 0.76 (0.04)^***^, R^2^ = 0.370. **p < 0.01, ***p < 0.001.

### 4.4 Intervening and sequential associations

The three structural equations are reported in Table 4. Engagement was positively associated with social presence (*b* = 0.61) and with perceived epistemic value (b = 0.44); social presence was positively associated with perceived epistemic value (*b* = 0.32); and both social presence (*b* = 0.21) and perceived epistemic value (*b* = 0.39) were positively associated with enjoyment (all *p* < 0.001). The bootstrap indirect association through social presence was reliable at the mean of ambiguity tolerance, indirect = 0.13, 95% bootstrap CI [0.09, 0.17], supporting H2; the indirect association through perceived epistemic value was reliable, indirect = 0.17, 95% CI [0.13, 0.22], supporting H3; and the serial indirect association through social presence and then perceived epistemic value was reliable, indirect = 0.08, 95% CI [0.06, 0.10], supporting H4. Conditional indirect associations at low, mean, and high ambiguity tolerance are reported in Table 5.

**Table 5:** Conditional indirect associations at levels of ambiguity tolerance.

Pathway	AT level	Estimate	95% bootstrap CI
Through social presence	Low (−1 SD)	0.169	[0.119, 0.224]
	Mean	0.129	[0.090, 0.170]
	High (+1 SD)	0.089	[0.057, 0.125]
Through perceived epistemic value	Low (−1 SD)	0.217	[0.165, 0.277]
	Mean	0.173	[0.133, 0.218]
	High (+1 SD)	0.129	[0.085, 0.176]
Serial (SP → PEV)	Low (−1 SD)	0.098	[0.072, 0.129]
	Mean	0.075	[0.055, 0.097]
	High (+1 SD)	0.051	[0.035, 0.071]

5,000 percentile bootstrap resamples. AT, ambiguity tolerance. The index of moderated serial mediation was −0.027, 95% CI [−0.041, −0.015].

### 4.5 Moderation by ambiguity tolerance

**Figure 2:**
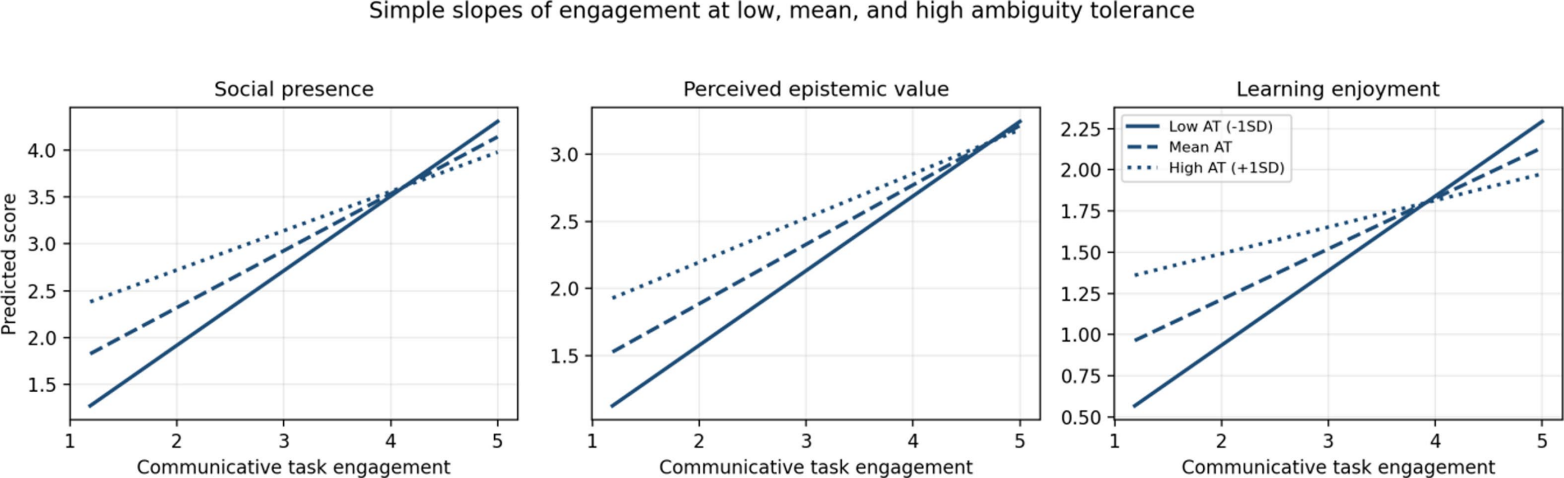
Simple slopes.

The product of engagement and ambiguity tolerance was negative and significant in all three equations (Table 4): for social presence, *b* = −0.22, *p* < 0.001; for perceived epistemic value, *b* = −0.13, *p* = 0.002; and for enjoyment, *b* = −0.17, *p* < 0.001. In each case the engagement-related association was stronger at lower ambiguity tolerance, supporting H5a, H5b, and H5c. The compensatory form of this moderation is evident in Table 5: the indirect association through social presence, for example, fell from 0.17 at low ambiguity tolerance to 0.09 at high ambiguity tolerance. To aid interpretation, simple slopes of engagement were computed at one standard deviation below the mean, the mean, and one standard deviation above (the standard deviation of ambiguity tolerance was 0.87), with standard errors obtained from the heteroscedasticity-consistent coefficient covariance via the delta method (Figure 2). For social presence, the simple slope of engagement was 0.80 (SE = 0.05) at low, 0.61 (SE = 0.04) at mean, and 0.42 (SE = 0.06) at high ambiguity tolerance; for perceived epistemic value, 0.56, 0.44, and 0.33; and for learning enjoyment, 0.45, 0.31, and 0.16. Every simple slope was positive and significant (all *p* < 0.001) and weaker at higher ambiguity tolerance, consistent with the compensatory pattern.

### 4.6 Conditional serial indirect association

The serial indirect association through social presence and perceived epistemic value was stronger at lower ambiguity tolerance—0.10, 95% CI [0.07, 0.13] at one standard deviation below the mean; 0.08, 95% CI [0.06, 0.10] at the mean; and 0.05, 95% CI [0.04, 0.07] at one standard deviation above (Table 5)—and the index of moderated serial mediation was negative and reliable, index = −0.03, 95% bootstrap CI [−0.04, −0.01], supporting H6. The index of moderated serial mediation is the formal test that the serial indirect association differs across levels of ambiguity tolerance; because its confidence interval excludes zero, the decline from lower to higher ambiguity tolerance is statistically reliable. A direct contrast confirmed this: the serial indirect association was reliably larger at low than at high ambiguity tolerance, difference = 0.046, 95% bootstrap CI [0.027, 0.071]. Substantively, this index indicates that at each one-unit-higher level of ambiguity tolerance, the serial indirect association through social presence and perceived epistemic value was smaller by approximately 0.03 units. This pattern suggests that the relational-then-evaluative pathway linking engaged practice to enjoyment was strongest among learners lower in ambiguity tolerance; across the conditional estimates reported in Table 5, the serial indirect association declined from 0.098 at low ambiguity tolerance to 0.051 at high ambiguity tolerance.

### 4.7 Interaction-mode differences in the moderation pattern

The three-way interaction of engagement, ambiguity tolerance, and interaction mode was examined in each equation (Table 6). It was negative and significant for social presence (*b* = −0.28, *p* = 0.001) and for perceived epistemic value (*b* = −0.26, *p* = 0.002) but did not reach significance for the direct enjoyment equation (*b* = −0.12, *p* = 0.088). The stronger compensatory pattern in richer interaction was thus evident for the social-presence and epistemic-value associations and not for the direct association with enjoyment. H7 was partially supported.

**Table 6:** Three-way interaction of engagement, ambiguity tolerance, and interaction mode.

Outcome equation	b	SE	*p*	95% CI
Social presence	−0.283	0.087	0.001	[−0.453, −0.114]
Perceived epistemic value	−0.260	0.084	0.002	[−0.424, −0.095]
Learning enjoyment	−0.122	0.072	0.088	[−0.263, 0.018]

Coefficients for the engagement × ambiguity tolerance × interaction-mode term, with HC3 standard errors. Interaction mode coded 0 = text-based only, 1 = richer voice-based/multimodal.

### 4.8 Interaction-mode differences in the conditional serial indirect association

The index of moderated serial mediation differed across interaction modes (Table 7). It was not statistically reliable in the text-based group, index = −0.002, 95% CI [−0.019, 0.010], but was reliable in the richer voice-based or multimodal interaction group, index = −0.037, 95% CI [−0.056, −0.021]. The bootstrapped difference between the two group-specific indices was also reliable, difference = −0.035, 95% CI [−0.057, −0.012]. This between-group difference supported a moderated moderated serial mediation pattern.

**Table 7:** Moderated serial mediation and conditional serial associations by interaction mode.

Interaction mode	Index of moderated serial mediation [95% CI]	Serial indirect at low / mean / high AT
Text-based only (*n* = 271)	−0.002 [−0.019, 0.010]	0.054/0.052/0.050
Richer voice-based/multimodal (*n* = 509)	−0.037 [−0.056, −0.021]	0.111/0.078/0.045
Difference (richer − text)	−0.035 [−0.057, −0.012]	—

5,000 percentile bootstrap resamples. AT, ambiguity tolerance.

The conditional serial indirect associations clarified the form of this pattern. In the richer-interaction group, the serial indirect association decreased from 0.111 at low ambiguity tolerance to 0.078 at the mean and 0.045 at high ambiguity tolerance. In the text-based group, the corresponding estimates were comparatively stable across ambiguity-tolerance levels, 0.054, 0.052, and 0.050, respectively. Thus, the stronger conditional serial indirect association in the richer-interaction group was mainly evident at lower and mean levels of ambiguity tolerance, rather than uniformly across all levels. H8 was supported with this qualification.

### 4.9 Sensitivity analysis

To verify that the results did not depend on conceptual overlap between social-interactive engagement and social presence, the model was re-estimated with the social-interactive engagement subscale removed from the engagement composite (Table 8; see also Supplementary Appendix G). The path estimates were comparable—engagement → social presence *b* = 0.56, social presence → perceived epistemic value *b* = 0.33, perceived epistemic value → enjoyment *b* = 0.40—and the index of moderated serial mediation remained negative and reliable, index = −0.02, 95% CI [−0.04, −0.01]. The pattern was therefore robust to this alternative operationalization of engagement. The results were also robust to the treatment of missing data. Re-estimating the model on complete cases only (*N* = 585, after listwise deletion of the 195 retained cases with any missing focal response) and under multiple imputation by chained equations (20 imputations) reproduced the substantive pattern; the index of moderated serial mediation was −0.024 under complete cases and −0.027 under pooled multiple imputation, against −0.027 in the main analysis. This sensitivity analysis further suggests that the structural pattern was not driven by overlap between social-interactive engagement and social presence.

**Table 8:** Sensitivity analysis.

Association	Estimate	95% CI
Engagement (reduced) → social presence	0.555	[0.481, 0.629]
Social presence → perceived epistemic value	0.332	[0.270, 0.394]
Perceived epistemic value → learning enjoyment	0.396	[0.330, 0.463]
Index of moderated serial mediation	−0.023	[−0.037, −0.011]

### 4.10 Diagnostics and summary of hypothesis tests

Variance inflation factors for the substantive predictors ranged from 1.06 to 1.89 (Table 9), and those for the covariates did not exceed 2.19, all well below conventional thresholds, indicating no multicollinearity concern. Table 10 summarizes the outcomes of the hypothesis tests.

**Table 9:** Variance inflation factors.

Predictor	VIF
Engagement (CTE)	1.69
Ambiguity tolerance (AT)	1.21
Engagement × AT	1.06
Social presence	1.69
Perceived epistemic value	1.89
Gender (covariate)	1.09–1.10
Self-rated proficiency (covariate)	1.69
AIGC-use frequency (covariate)	1.72–2.19

**Table 10:** Summary of hypothesis tests.

Hypothesis	Outcome
H1: engagement → enjoyment (positive)	Supported
H2: social presence as an intervening variable	Supported
H3: perceived epistemic value as an intervening variable	Supported
H4: serial pathway (SP → PEV)	Supported
H5a–c: AT negatively moderates engagement paths	Supported
H6: AT-moderated serial indirect (stronger at lower AT)	Supported
H7: stronger moderation in richer interaction	Partially supported (SP, PEV; not LE)
H8: conditional serial indirect stronger in richer interaction	Supported with qualification

## 5 Discussion

### 5.1 Overview of findings

This study examined how, for whom, and through which channel AIGC-supported communicative task engagement is associated with learning enjoyment. The pattern of results was consistent with the proposed account. Engagement was positively associated with enjoyment, and the results were consistent with an indirect association through social presence and then perceived epistemic value. The engagement-related associations were stronger for learners lower in ambiguity tolerance, and the conditional serial indirect association was likewise more pronounced at lower ambiguity tolerance. Subgroup analyses by interaction mode further qualified this pattern: the stronger compensatory moderation in the richer-interaction subgroup was reliable for the social-presence and perceived-epistemic-value associations but not for the direct association with enjoyment, and the conditional serial indirect association was reliable, and more clearly stronger at lower ambiguity tolerance, only in the richer-interaction subgroup. Because the design is cross-sectional and the measures self-reported, these findings describe a structure of associations consistent with the proposed ordering; they do not establish temporal sequence or causation.

### 5.2 Theoretical contributions

The first contribution is theoretical integration. Enjoyment in language learning has developed largely within the positive-psychology tradition (Dewaele and Li, 2020; Dewaele and MacIntyre, 2014; Wang et al., 2021), while control-value theory locates the proximal sources of such emotions in appraisals of value and control (Pekrun, 2006) but was formulated for classroom achievement and says little about communication. Reading control-value theory together with an account of social presence (Kreijns et al., 2003; Makransky et al., 2017) specifies a route by which a relational experience and an evaluative appraisal jointly link engagement to enjoyment, and the fit of the proposed ordering—social presence first, perceived epistemic value second—is consistent with evidence that social presence accompanies value perceptions in chatbot-assisted English learning (Hong et al., 2024).

The findings also provide measurement-level support for distinguishing engagement as an in-task process from perceived epistemic value as an evaluative appraisal. Cognitive engagement refers to effortful processing during the task, whereas perceived epistemic value refers to the learner’s judgment that the activity advanced language knowledge and understanding. In the present measurement model, perceived-epistemic-value items and cognitive-engagement items loaded on separate factors, and no cross-loading reached 0.30. This pattern supports treating the two constructs as empirically distinct in this study, while further validation in other samples remains desirable.

A second contribution concerns scope. Task engagement has been studied mainly in classroom activities with teachers and peers (Hiver et al., 2024; Mercer and Dörnyei, 2020; Philp and Duchesne, 2016; Qiu, 2025); the present findings are consistent with that conception and extend it to communicative practice in which the interlocutor is a generative system, where behavioral, cognitive, agentic, and social-interactive involvement remained associated with the quality of the experience (Zare and Derakhshan, 2025). For research on foreign language enjoyment, the study answers a specific critique—that enjoyment is frequently treated as a static, individual, classroom-bound trait—by modeling it as part of an interactional and evaluative process in a learner–AI context (Wu and Kabilan, 2025).

A third contribution is conceptual precision around value. Much work on AI-assisted learning frames the worth of these tools in terms of usefulness or the pleasure of using them (Mohebbi, 2025; Wang et al., 2026). The present study instead isolates perceived epistemic value—a judgment that an activity advanced knowledge and understanding (Peels, 2018)—holding it apart from cognitive engagement as an in-task process and from hedonic value as the pleasure of the activity (Hong et al., 2016; Philp and Duchesne, 2016). That this appraisal was associated with engagement and, in turn, with enjoyment is consistent with the situated, content-bearing view of value in expectancy-value theory (Eccles and Wigfield, 2020).

A fourth contribution lies in the boundary conditions, where the study links a learner difference to a feature of the channel. Ambiguity tolerance is often treated as an adaptive trait (Chu et al., 2015; McLain, 2009); here it operated as a compensatory boundary condition, with engagement more strongly associated with the relational and evaluative correlates for learners lower in ambiguity tolerance, consistent with its negative moderation of the engagement–effectiveness association in technology-supported learning (Zhang, 2024). Extending this, the compensatory pattern was more pronounced in richer, voice-based or multimodal interaction for the social-presence and perceived-epistemic-value associations, consistent with evidence that modes of multimedia input differ in effectiveness and that richer input is associated with deeper processing and stronger engagement (Wang et al., 2026; Zhang and Zou, 2022), and answering the call to examine how learner factors interact with features of multimedia input (Zhang and Zou, 2022). That the three-way pattern did not reach significance for the direct association with enjoyment suggests that the subgroup difference is concentrated mainly in the relational and evaluative associations, with the unmediated engagement–enjoyment association less clearly differentiated by interaction mode. In other words, the interaction-mode difference appears to be most evident in the relational and evaluative components of the model, while the residual direct association between engagement and enjoyment was comparatively similar across the two interaction-mode subgroups.

The moderating role of ambiguity tolerance is also meaningful when viewed alongside recent AI-IDLE research. Conversational GenAI tools may provide a relatively safe and non-judgmental space for communicative practice (Wang et al., 2024), but AI-IDLE can also place cognitive and strategic demands on learners because they must formulate prompts, negotiate meaning, and evaluate open-ended AI output without direct teacher structuring (Liu et al., 2024). In this sense, AIGC-supported practice may reduce some forms of social pressure while introducing epistemic and cognitive uncertainty. The present findings are consistent with this tension: engagement was more strongly associated with the relational and evaluative components of enjoyment among learners lower in ambiguity tolerance, suggesting a compensatory pattern under conditions of uncertainty.

This pattern converges with and extends Zhang (2024), who found that ambiguity tolerance negatively moderated the engagement–effectiveness association in metaverse-based language learning. The present study observes the same compensatory direction for an affective outcome and specifies a relational and evaluative pathway, together with a channel-level boundary condition. Because the proposed ordering rests on a socio-emotional-first logic rather than on features unique to AIGC, it may also be relevant to other mediated communicative settings, a possibility for future study.

### 5.3 Practical contributions

The findings, though associational, bear on three practical problems. For sustaining voluntary practice, the results point away from access and toward involvement: enjoyment was associated with the depth of engagement and with the sense that the activity was responsive and worthwhile, which suggests designing tasks that call for sustained dialogue, clarification, feedback, and reflection, going beyond answer retrieval. For making solitary practice feel meaningful, the place of social presence suggests building tasks around communicative behaviors—turn-taking, meaning negotiation, feedback-seeking—that invite learners to treat the system as a communicative partner while remaining aware of the limits of generated output. For supporting learners less comfortable with uncertainty, the moderation and channel findings suggest that uncertainty-averse learners in particular may benefit from tasks that make uncertainty manageable—clear goals, structured prompts, explicit feedback cycles—and that, for these learners, richer voice-based or multimodal interaction may provide more of the contextual support that engaged interaction draws on. Concretely, such tasks might include information-gap role-plays that require clarifying questions (for example, completing a travel booking from incomplete details), goal-directed negotiation tasks with a turn limit and an explicit feedback step, and short reflection prompts in which the learner restates in the target language what the exchange clarified. Richer voice-based or multimodal versions of these tasks, however, depend on reliable speech or multimodal processing, acceptable latency, adequate devices and bandwidth, and privacy safeguards. Because no intervention was tested, these are directions for design to be evaluated, not established prescriptions.

These design implications also connect with AI-IDLE research on continuance intention. In self-directed AI-assisted language learning, enjoyment is not merely a by-product of task performance but one condition associated with learners’ willingness to continue using AI tools after the initial novelty has faded. Fan and Zhang (2024), for example, reported that foreign language enjoyment was linked to continuance intention in AI-assisted EFL learning. The present findings extend this continuance-oriented view by indicating what kind of practice may support enjoyment: not passive answer retrieval, but sustained, agentic participation in which learners steer the exchange by setting goals, requesting clarification, asking follow-up questions, and negotiating meaning. Thus, AIGC-supported tasks should invite learners to actively shape the conversation rather than simply consume generated text; whether such designs increase long-term voluntary practice should be tested in future intervention studies.

### 5.4 Limitations and future research

Several limitations bound these conclusions. The design is cross-sectional and the measures self-reported, so the results describe associations consistent with the proposed ordering and cannot demonstrate temporal sequence or causation; longitudinal, experience-sampling, and experimental designs are needed. Interaction mode was self-reported and not assigned, so the channel-related differences should be interpreted as subgroup patterns rather than modality effects, and they await controlled comparison of text-, voice-, and multimodal tasks; relatedly, only configural invariance across modes was established here, and formal metric and scalar invariance should be tested before strong cross-mode claims are made. The perceived epistemic value scale was newly assembled and contextualized for the present study. Although the items were grounded in prior conceptual and operational work and reviewed during the bilingual translation and back-translation procedure for semantic equivalence and contextual appropriateness, no separate expert-panel content-validity index was conducted. Therefore, the scale requires further validation across samples, languages, and AIGC systems. Because all focal variables—engagement, social presence, perceived epistemic value, learning enjoyment, and ambiguity tolerance—were measured with the same self-report questionnaire, common method bias is a concern. The diagnostics reported above did not indicate a dominant common method factor: Harman’s single-factor test attributed only 32.78% of the variance to the first factor, and a single-factor confirmatory model fit poorly. In addition, the total engagement–enjoyment association declined substantially once social presence and perceived epistemic value were entered, which is less consistent with a purely uniform method-driven inflation of associations. These remain diagnostics rather than remedies, however, and common method bias cannot be excluded under a cross-sectional self-report design; multi-source, multi-method, or marker-variable approaches are recommended for future replication. The absence of a significant three-way interaction in the direct enjoyment equation suggests that channel richness may matter more for the relational and evaluative components of the process than for the residual engagement–enjoyment association. The weaker conditional pattern at higher ambiguity tolerance is consistent with the compensatory account, since learners comfortable with uncertainty rely less on active engagement to stabilize the exchange or buffer against psychological discomfort, leaving little for the pathway to compensate (Chu et al., 2015; Zhang, 2024). Furthermore, a ceiling effect at the upper end of ambiguity tolerance may also contribute to this non-significance, a possibility for future work to examine directly. Finally, the sample comprised learners who use AIGC for communicative practice; generalization to other populations and settings is a matter for further study.

Furthermore, regarding the directionality of the serial sequence, a reverse ordering—in which an appraisal of epistemic value precedes a sense of social presence—is conceivable. The present ordering is adopted on theoretical grounds, which posit that socio-emotional perceptions of an interactive environment typically condition subsequent cognitive appraisals (Hong et al., 2024; Kreijns et al., 2003). However, because a cross-sectional design cannot establish temporal sequence, the alternative ordering remains an open question for future longitudinal and experimental designs.

## 6 Conclusion

The study offers an associational account of learning enjoyment in AIGC-supported communicative practice and finds a pattern consistent with the proposed model: engagement was associated with enjoyment, this association was statistically linked to social presence and perceived epistemic value, and the strength of the engagement-related associations varied with the learner’s tolerance of ambiguity and, for the relational and evaluative correlates, with interaction-mode subgroup. Because the design is correlational, these patterns describe associations consistent with the proposed ordering and do not establish temporal sequence or causation. Its contribution is to locate the value of AIGC-supported communicative practice not in access to the tool alone but in the affective and relational experience associated with it, and to describe learner-side and channel-side conditions under which this association appears stronger.

## Data Availability

The raw data supporting the conclusions of this article will be made available by the authors, without undue reservation.

## References

[ref1] Alhusaiyan E. (2025). A systematic review of current trends in artificial intelligence in foreign language learning. Saudi J. Lang. Stud. 5, 1–16. doi: 10.1108/SJLS-07-2024-0039

[ref2] Annamalai N. Rashid R. A. Hashmi U. M. Mohamed M. Alqaryouti M. H. Sadeq A. E. (2023). Using chatbots for English language learning in higher education. Comput. Educ.: Artif. Intell. 5:100153. doi: 10.1016/j.caeai.2023.100153

[ref3] Aslan E. Thompson A. S. (2021). The interplay between learner beliefs and foreign language anxiety: insights from the Turkish EFL context. Lang. Learn. J. 49, 189–202. doi: 10.1080/09571736.2018.1540649

[ref4] Aydın S. Tekin I. Akkaş F. D. (2024). Construction and validation of the foreign language learning enjoyment scale. Psychol. Sch. 61, 657–670. doi: 10.1002/pits.23076

[ref5] Cai Y. Xing K. (2025). Examining the mediation of engagement between self-efficacy and language achievement. J. Multiling. Multicult. Dev. 46, 893–905. doi: 10.1080/01434632.2023.2217801

[ref6] Çakmak F. (2022). Chatbot-human interaction and its effects on EFL students’ L2 speaking performance and anxiety. Novitas-ROYAL (Research on Youth and Language) 16, 113–131.

[ref7] Chen L. (2023). Learner autonomy and English achievement in Chinese EFL undergraduates: the mediating role of ambiguity tolerance and foreign language classroom anxiety. Lang. Learn. High. Educ. 13, 295–308. doi: 10.1515/cercles-2023-2001

[ref8] Chen A. Zhang Y. Jia J. Liang M. Cha Y. Lim C. P. (2025). A systematic review and meta-analysis of AI-enabled assessment in language learning: design, implementation, and effectiveness. J. Comput. Assist. Learn. 41:e13064. doi: 10.1111/jcal.13064

[ref9] Chu W. H. Lin D. Y. Chen T. Y. Tsai P. S. Wang C. H. (2015). The relationships between ambiguity tolerance, learning strategies, and learning Chinese as a second language. System 49, 1–16. doi: 10.1016/j.system.2014.10.015

[ref10] Daft R. L. Lengel R. H. (1986). Organizational information requirements, media richness and structural design. Manag. Sci. 32, 554–571. doi: 10.1287/mnsc.32.5.554, 19642375

[ref11] Dewaele J.-M. Li C. (2020). Emotions in second language acquisition: a critical review and research agenda. Foreign Lang. World 196, 34–49.

[ref12] Dewaele J.-M. MacIntyre P. D. (2014). The two faces of Janus? Anxiety and enjoyment in the foreign language classroom. Stud. Second Lang. Learn. Teach. 4, 237–274. doi: 10.14746/ssllt.2014.4.2.5

[ref13] Eccles J. S. Wigfield A. (2020). From expectancy-value theory to situated expectancy-value theory: a developmental, social cognitive, and sociocultural perspective on motivation. Contemp. Educ. Psychol. 61:101859. doi: 10.1016/j.cedpsych.2020.101859

[ref14] Egbert J. Shahrokni S. A. Zhang X. Abobaker R. Bantawtook P. He H. . (2021). Language task engagement: an evidence-based model. TESL-EJ 24.

[ref15] Ely C. M. (1995). “Tolerance of ambiguity and the teaching of ESL,” in Learning styles in the ESL/EFL Classroom, ed. Reid J. M. (New York: Heinle & Heinle), 87–95.

[ref16] Fan J. Zhang Q. (2024). From literacy to learning: the sequential mediation of attitudes and enjoyment in AI-assisted EFL education. Heliyon 10:e37158. doi: 10.1016/j.heliyon.2024.e37158, 39286160 PMC11402731

[ref19] Hiver P. Al-Hoorie A. H. Vitta J. P. Wu J. (2024). Engagement in language learning: a systematic review of 20 years of research methods and definitions. Lang. Teach. Res. 28, 201–230. doi: 10.1177/13621688211001289

[ref20] Hong J.-C. Hwang M. Y. Tai K. H. Kuo Y. C. (2016). Crystallized intelligence affects hedonic and epistemic values to continue playing a game with saliency-based design. Comput. Educ. 95, 75–84. doi: 10.1016/j.compedu.2015.12.006

[ref21] Hong J.-C. Lin C.-H. Juh C.-C. (2024). Using a chatbot to learn English via charades: the correlates between social presence, hedonic value, perceived value, and learning outcome. Interact. Learn. Environ. 32, 6590–6606. doi: 10.1080/10494820.2023.2273485

[ref22] Kreijns K. Kirschner P. A. Jochems W. (2003). Identifying the pitfalls for social interaction in computer-supported collaborative learning environments: a review of the research. Comput. Hum. Behav. 19, 335–353. doi: 10.1016/S0747-5632(02)00057-2

[ref23] Lee S. Choe H. Zou D. Jeon J. (2025). Generative AI (GenAI) in the language classroom: a systematic review. Interact. Learn. Environ. 34, 1–25. doi: 10.1080/10494820.2025.2498537

[ref24] Lei M. Zou Y. Wang X. (2026). Threat-related AI anxiety and engagement in live-streamed AI courses: a moderated serial mediation model of extrinsic motivation and teacher support. Front. Psychol. 17:1831785. doi: 10.3389/fpsyg.2026.1831785, 42137079 PMC13168054

[ref25] Li Y. Zhou X. Yin H.-B. Chiu T. K. F. (2025). Design language learning with artificial intelligence (AI) chatbots based on activity theory from a systematic review. Smart Learn. Environ. 12:24. doi: 10.1186/s40561-025-00379-0

[ref26] Lin Y. Zou Y. Wang X. (2026). Conditional trust pathways in live-streaming commerce: how consumer motivation influences responses to human and AI anchors. Front. Psychol. 17:1797803. doi: 10.3389/fpsyg.2026.1797803, 42157987 PMC13180573

[ref27] Liu G. L. Darvin R. Ma C. (2024). Exploring AI-mediated informal digital learning of English (AI-IDLE): a mixed-method investigation of Chinese EFL learners’ AI adoption and experiences. Comput. Assist. Lang. Learn.. Advance online publication. doi:doi: 10.1080/09588221.2024.2310288

[ref28] Makransky G. Lilleholt L. Aaby A. (2017). Development and validation of the multimodal presence scale for virtual reality environments: a confirmatory factor analysis and item response theory approach. Comput. Hum. Behav. 72, 276–285. doi: 10.1016/j.chb.2017.02.066

[ref29] McGrath C. Farazouli A. Cerratto-Pargman T. (2024). Generative AI chatbots in higher education: a review of an emerging research area. High. Educ. 89, 1533–1549. doi: 10.1007/s10734-024-01288-w

[ref30] McLain D. L. (2009). Evidence of the properties of an ambiguity tolerance measure: the multiple stimulus types ambiguity tolerance scale–II (MSTAT-II). Psychol. Rep. 105, 975–988. doi: 10.2466/PR0.105.3.975-988, 20099561

[ref31] Mercer S. Dörnyei Z. (2020). Engaging Language Learners in Contemporary Classrooms. Cambridge: Cambridge University Press.

[ref32] Mohebbi A. (2025). Enabling learner independence and self-regulation in language education using AI tools: a systematic review. Cogent Educ. 12:2433814. doi: 10.1080/2331186X.2024.2433814

[ref33] Peels R. (2018). Epistemic values in the humanities and in the sciences. Hist. Hum. 3, 89–107. doi: 10.1086/696304

[ref34] Pekrun R. (2006). The control-value theory of achievement emotions: assumptions, corollaries, and implications for educational research and practice. Educ. Psychol. Rev. 18, 315–341. doi: 10.1007/s10648-006-9029-9

[ref35] Philp J. Duchesne S. (2016). Exploring engagement in tasks in the language classroom. Annu. Rev. Appl. Linguist. 36, 50–72. doi: 10.1017/S0267190515000094

[ref36] Qiu X. (2025). Communicative task engagement in second language learning and teaching: a scoping review. Lang. Learn. J. 53, 819–844. doi: 10.1080/09571736.2025.2502733

[ref37] Reeve J. Tseng C.-M. (2011). Agency as a fourth aspect of students’ engagement during learning activities. Contemp. Educ. Psychol. 36, 257–267. doi: 10.1016/j.cedpsych.2011.05.002

[ref38] Şahin Kızıl A. Klimova B. Pikhart M. Parmaxi A. (2025). A systematic review of the recent research on the usefulness of chatbots for language education. J. Comput. Assist. Learn. 41:e70001. doi: 10.1111/jcal.70001

[ref39] Sweller J. (1988). Cognitive load during problem solving: effects on learning. Cogn. Sci. 12, 257–285. doi: 10.1207/s15516709cog1202_4

[ref40] Tantucci V. Wang A. Culpeper J. (2022). Reciprocity and epistemicity: on the (proto)social and cross-cultural ‘value’ of information transmission. J. Pragmat. 194, 54–70. doi: 10.1016/j.pragma.2022.04.012

[ref41] Wang Y. Derakhshan A. Zhang L. J. (2021). Researching and practicing positive psychology in second/foreign language learning and teaching: the past, current status, and future directions. Front. Psychol. 12:731721. doi: 10.3389/fpsyg.2021.731721, 34489835 PMC8417049

[ref42] Wang C. Du Y. Zou B. (2026). Learners’ acceptance and use of multimodal artificial intelligence (AI)-generated content in AI-mediated informal digital learning of English. Int. J. Appl. Linguist.. Advance online publication. doi:doi: 10.1111/ijal.12827

[ref43] Wang F. Wang X. (2025a). Let’s post more! The impact of foreign language social grooming on social media on learners’ enjoyment: a moderated mediation model. Front. Psychol. 16:1674786. doi: 10.3389/fpsyg.2025.1674786, 41132601 PMC12540393

[ref44] Wang F. Wang X. (2025b). The effect of social grooming via live photo-sharing on well-being: the mediating role of social capital and moderating role of the need for privacy. Front. Psychol. 16:1627455. doi: 10.3389/fpsyg.2025.1627455, 40718558 PMC12289657

[ref45] Wang Y. Zhang T. Yao L. Seedhouse P. (2025). A scoping review of empirical studies on generative artificial intelligence in language education. Innov. Lang. Learn. Teach., 1–28. doi: 10.1080/17501229.2025.2509759, 37339054

[ref46] Wang C. Zou B. Du Y. Wang Z. (2024). The impact of different conversational generative AI chatbots on EFL learners: an analysis of willingness to communicate, foreign language speaking anxiety, and self-perceived communicative competence. System 127:103533. doi: 10.1016/j.system.2024.103533

[ref47] Wiboolyasarin W. Wiboolyasarin K. Tiranant P. Jinowat N. Boonyakitanont P. (2025). AI-driven chatbots in second language education: a systematic review of their efficacy and pedagogical implications. Ampersand 14:100224. doi: 10.1016/j.amper.2025.100224

[ref48] Wu W. Kabilan M. K. (2025). Foreign language enjoyment in language learning from a positive psychology perspective: a scoping review. Front. Psychol. 16:1545114. doi: 10.3389/fpsyg.2025.1545114, 40391114 PMC12087380

[ref49] Yang T. Xu W. Li H. T. (2026). Self-efficacy and learning engagement in EFL context: a moderated mediation model of enjoyment, anxiety, and boredom. Acta Psychol. 264:106441. doi: 10.1016/j.actpsy.2026.106441, 41679149

[ref50] Yu M. Wang H. Xia G. (2022). The review on the role of ambiguity of tolerance and resilience on students' engagement. Front. Psychol. 12:828894. doi: 10.3389/fpsyg.2021.828894, 35095705 PMC8792790

[ref52] Zhang Q. (2024). Mediation/moderation effects of engagement, foreign language enjoyment, and ambiguity tolerance in metaverse-based foreign language learning. Int. J. Educ. Technol. High. Educ. 21:54. doi: 10.1186/s41239-024-00484-z

[ref53] Zhang Z. Gao X. Liu T. (2024). Enjoyment in foreign language learning: a systematic review. Heliyon 10:e37215. doi: 10.1016/j.heliyon.2024.e37215, 39296239 PMC11408776

[ref54] Zhang S. Liu X. (2025). Learner emotions in AI-assisted English as a second/foreign language learning: a systematic review of empirical studies. Front. Psychol. 16:1652806. doi: 10.3389/fpsyg.2025.1652806, 41112531 PMC12529940

[ref55] Zhang R. Zou D. (2022). A state-of-the-art review of the modes and effectiveness of multimedia input for second and foreign language learning. Comput. Assist. Lang. Learn. 35, 2790–2816. doi: 10.1080/09588221.2021.1896555

