# Supplementary material for: When AI becomes a language partner: a moderated serial mediation model of AIGC-supported communicative task engagement and learning enjoyment
Source: Front Psychol. 2026 Jul 22;17:1903870. doi: 10.3389/fpsyg.2026.1903870 (PMC13439968; doi:10.3389/fpsyg.2026.1903870)
Supplement: Supplementary file 1 [file Data_sheet_1.pdf]

Supplementary Material

1 Appendix A. Abbreviations, variables, and coding

Table A1 defines the construct abbreviations and the role of each focal variable in the model. Table A2 reports the coding of background and heterogeneity variables used in the dataset and analyses.

Table A1

Construct abbreviations

Abbreviation	*Meaning*
AIGC	AI-generated content; generative AI tools used for communicative language practice
CTE	AIGC-supported communicative task engagement; higher-order construct measured by BE, CE, AE, and SIE
BE	Behavioral engagement
CE	Cognitive engagement
AE	Agentic engagement
SIE	Social-interactive engagement
SP	Social presence
PEV	Perceived epistemic value
LE	Learning enjoyment
AT	Ambiguity tolerance
IM / Mode	Interaction mode; raw three-category item recoded as text-based only vs richer interaction for H7-H8

Table A2

Coding of background and heterogeneity variables

Variable	*Question / variable label*	*Codes and labels*
Q1_Gender	Gender	1 = Male; 2 = Female; 3 = Prefer not to say
Q2_Age	Age band	Reported as adult bands in Appendix B; no participants below 18 were retained.
Q3_EduLevel	Educational level	1 = High school or below; 2 = Undergraduate (Bachelor's); 3 = Postgraduate (Master's); 4 = Doctorate (PhD) or above
Q4_Grade	Grade/year of study	1 = Year 1; 2 = Year 2; 3 = Year 3; 4 = Year 4; 5 = Graduate student
Q5_Major	Major field	1 = Language, linguistics, literature; 2 = STEM; 3 = Social sciences or humanities; 4 = Business or economics; 5 = Other
Q6_TargetLang	Target language	1 = English; 2 = Japanese; 3 = Korean; 4 = European languages; 5 = Other

Q7_YearsLearning	Years learning target language	1 = Less than 1 year; 2 = 1 to 3 years; 3 = 3 to 5 years; 4 = More than 5 years
Q8_Proficiency	Self-rated proficiency	1 = Beginner; 2 = Intermediate; 3 = Advanced
Q9_TestScore	Standardized test-score band	1 = No official score; 2 = Basic (CEFR A1/A2); 3 = Independent (CEFR B1/B2); 4 = Proficient (CEFR C1/C2)
Q10_FreqRealComm	Frequency of real-life communication	1 = Rarely or never; 2 = A few times a month; 3 = A few times a week; 4 = Daily
Q11_FreqAIGC_Gen	Frequency of general AIGC use	1 = Rarely; 2 = A few times a month; 3 = A few times a week; 4 = Daily
Q12_FreqAIGC_Task	Frequency of AIGC use for communicative tasks	1 = Rarely; 2 = A few times a month; 3 = A few times a week; 4 = Daily
Q13_AIGC_Tool	Primary AIGC tool	1 = ChatGPT; 2 = Kimi; 3 = Doubao; 4 = ERNIE Bot; 5 = Other
Q14_Purpose	Primary purpose of AIGC use	1 = Writing and grammar; 2 = Speaking and role-play; 3 = Vocabulary and translation; 4 = Unscripted chatting
Q15_InteractionMode	Primary interaction mode (raw)	1 = Text-based only; 2 = Text + voice; 3 = Text + voice + visual/video or other multimodal cues
ModeRich	Interaction-mode group used in H7-H8	0 = Text-based only; 1 = Richer interaction (text + voice or text + voice + visual/video/multimodal)
Q16_LearningContext	Learning context	1 = Primarily in class; 2 = Primarily outside class; 3 = Equally both

Note. The raw interaction-mode item contained three response categories: text-based only, text + voice, and text + voice + visual/video or other multimodal cues. For H7-H8, code 1 was retained as the text-based-only group, whereas codes 2 and 3 were combined as the richer-interaction group because both involved non-textual communicative cues. This recoding was used only for the interaction-mode heterogeneity analyses; the raw three-category distribution is reported in Appendix B.

2 Appendix B. Demographic and background profile of the analytic sample

Table B1 reports frequencies and percentages for the final analytic sample (N = 780). Primary AIGC tool: ChatGPT 302 (38.7%), Kimi 152 (19.5%), ERNIE Bot 125 (16.0%), Doubao 119 (15.3%), Other 82 (10.5%).

Table B1

Frequencies and percentages for the final analytic sample

Variable	*Category*	*n*	*%*
Age band	18-22	468	60.0
Age band	23-26	227	29.1
Age band	27 or above	85	10.9

Gender	Male	322	41.3
Gender	Female	412	52.8
Gender	Prefer not to say	46	5.9
Educational level	High school or below	47	6.0
Educational level	Undergraduate (Bachelor's)	474	60.8
Educational level	Postgraduate (Master's)	221	28.3
Educational level	Doctorate (PhD) or above	38	4.9
Grade/year of study	Year 1	109	14.0
Grade/year of study	Year 2	141	18.1
Grade/year of study	Year 3	216	27.7
Grade/year of study	Year 4	78	10.0
Grade/year of study	Graduate student	236	30.3
Major field	Language, linguistics, literature	229	29.4
Major field	STEM	201	25.8
Major field	Social sciences or humanities	139	17.8
Major field	Business or economics	118	15.1
Major field	Other	93	11.9
Target language	English	452	57.9
Target language	Japanese	125	16.0
Target language	Korean	82	10.5
Target language	European languages	88	11.3
Target language	Other	33	4.2
Years learning target language	Less than 1 year	67	8.6
Years learning target language	1 to 3 years	245	31.4
Years learning target language	3 to 5 years	272	34.9
Years learning target language	More than 5 years	196	25.1
Self-rated proficiency	Beginner	139	17.8
Self-rated proficiency	Intermediate	492	63.1
Self-rated proficiency	Advanced	149	19.1
Standardized test-score band	No official score	102	13.1
Standardized test-score band	Basic (CEFR A1/A2)	187	24.0
Standardized test-score band	Independent (CEFR B1/B2)	338	43.3
Standardized test-score band	Proficient (CEFR C1/C2)	153	19.6
Frequency of real-life communication	Rarely or never	164	21.0
Frequency of real-life communication	A few times a month	303	38.8
Frequency of real-life communication	A few times a week	223	28.6
Frequency of real-life	Daily	90	11.5

communication			
Frequency of general AIGC use	Rarely	62	7.9
Frequency of general AIGC use	A few times a month	204	26.2
Frequency of general AIGC use	A few times a week	358	45.9
Frequency of general AIGC use	Daily	156	20.0
Frequency of AIGC use for communicative tasks	Rarely	120	15.4
Frequency of AIGC use for communicative tasks	A few times a month	253	32.4
Frequency of AIGC use for communicative tasks	A few times a week	289	37.1
Frequency of AIGC use for communicative tasks	Daily	118	15.1
Primary AIGC tool	ChatGPT	302	38.7
Primary AIGC tool	Kimi	152	19.5
Primary AIGC tool	Doubao	119	15.3
Primary AIGC tool	ERNIE Bot	125	16.0
Primary AIGC tool	Other	82	10.5
Primary purpose of AIGC use	Writing and grammar	244	31.3
Primary purpose of AIGC use	Speaking and role-play	276	35.4
Primary purpose of AIGC use	Vocabulary and translation	149	19.1
Primary purpose of AIGC use	Unscripted chatting	111	14.2
Primary interaction mode (raw)	Text-based only	271	34.7
Primary interaction mode (raw)	Text + voice	247	31.7
Primary interaction mode (raw)	Text + voice + visual/video or other multimodal cues	262	33.6
Interaction-mode group used in H7-H8	Text-based only	271	34.7
Interaction-mode group used in H7-H8	Richer interaction (text + voice or text + voice + visual/video/multimodal)	509	65.3
Learning context	Primarily in class	149	19.1
Learning context	Primarily outside class	399	51.2
Learning context	Equally both	232	29.7

Note. Age is reported as adult bands; no participants below 18 were retained in the analytic sample. Percentages may not sum to 100 due to rounding.

3 Appendix C. Measurement items, item-level statistics, and EFA primary loadings

Table C1 reports the full item wording, descriptive statistics, corrected item-total correlations, and rotated primary loadings from the exploratory factor analysis. All focal items were rated from 1 (strongly disagree) to 5 (strongly agree).

Table C1

Item wording, descriptive statistics, corrected item-total correlations, and rotated primary loadings

Item	*Construct*	*Item wording*	*M*	*SD*	*Item-total r*	*EFA loading*
B1	BE	I work hard during AIGC-supported communicative tasks. / 在 AIGC 支持的交际任务中，我会努力完成任务。	3.00	1.02	0.70	0.65
B2	BE	I stay focused and avoid distraction during AIGC-supported communicative tasks. / 在 AIGC 支持的交际任务中，我会保持专注，避免分心。	3.19	1.08	0.71	0.79
B3	BE	I put in the effort needed to complete AIGC-supported communicative tasks well. / 在 AIGC 支持的交际任务中，我会投入把任务做好所需要的努力。	3.00	1.01	0.71	0.82
B4	BE	I try to do the task thoroughly rather than just getting it done. / 在 AIGC 支持的交际任务中，我会力求把任务做充分，而不只是完成了事。	3.56	1.10	0.77	0.88
B5	CE	During AIGC-supported communicative tasks, I try to put key language points into my own words. / 在 AIGC 支持的交际任务中，我会尝试用自己的话表达关键的语言点。	3.72	1.10	0.63	0.70
B6	CE	During AIGC-supported communicative tasks, I try to connect the AI's responses to what I already know. / 在 AIGC 支持的交际任务中，我会尝试把 AI 的回应与我已有的知识联系起来。	3.79	1.13	0.65	0.62
B7	CE	During AIGC-supported communicative tasks, I think of my own examples to work out how to use the target language. / 在 AIGC 支持的交际任务中，我会尝试举出自己的例子，以弄清如何使用目标语。	3.68	1.13	0.71	0.75
B8	CE	During AIGC-supported communicative tasks, I review the AI's responses and question myself about them. / 在 AIGC 支持的交际任务中，我会回顾 AI 的回应，并就这些回应进行自我提问。	3.68	1.13	0.73	0.88

B9	AE	During AIGC-supported communicative tasks, I tell the AI what I want or need to practice. / 在 AIGC 支持的交际任务中，我会告诉 AI 我想练习或需要练习什么。	3.99	1.10	0.58	0.65
B10	AE	During AIGC-supported communicative tasks, I let the AI know the topics or situations I am interested in. / 在 AIGC 支持的交际任务中，我会让 AI 知道我感兴趣的话题或情境。	3.56	1.12	0.61	0.65
B11	AE	During AIGC-supported communicative tasks, I express my own preferences and opinions to steer the interaction. / 在 AIGC 支持的交际任务中，我会表达自己的偏好和看法，以引导互动的方向。	3.06	1.03	0.63	0.64
B12	AE	During AIGC-supported communicative tasks, I ask the AI to focus on what I most want to improve. / 在 AIGC 支持的交际任务中，我会要求 AI 着重处理我最想提高的方面。	3.16	1.07	0.64	0.75
B13	SIE	During AIGC-supported communicative tasks, I ask the AI to help me work through the task. / 在 AIGC 支持的交际任务中，我会请 AI 帮助我推进任务。	2.99	1.03	0.73	0.79
B14	SIE	During AIGC-supported communicative tasks, I build on the AI's responses to take the conversation further. / 在 AIGC 支持的交际任务中，我会借助 AI 的回应把对话继续推进。	3.86	1.13	0.60	0.71
B15	SIE	During AIGC-supported communicative tasks, I ask the AI for feedback on my language use. / 在 AIGC 支持的交际任务中，我会请 AI 对我的语言使用给予反馈。	2.94	1.01	0.72	0.79
B16	SIE	During AIGC-supported communicative tasks, I ask the AI follow-up questions to develop the exchange. / 在 AIGC 支持的交际任务中，我会向 AI 追问，以展开彼此的交流。	3.54	1.14	0.70	0.76
C1	SP	When I interact with the AIGC system, I feel as if another communicative partner is present. / 与 AIGC 系统互动时，我感觉仿佛有另一位交际伙伴在场。	3.29	1.11	0.64	0.76
C2	SP	The AIGC system seems aware of what I am communicating. / 与 AIGC 系统互动时，我感觉它能意识到我所表达的内容。	3.36	1.08	0.66	0.76
C3	SP	The AIGC system responds to me in a socially meaningful way. / AIGC 系统能以具有社会意义的方式回应我。	3.16	1.07	0.67	0.79

C4	SP	I feel I am communicating with a partner rather than simply operating a computer system. / 我感觉自己是在与一位交际伙伴交流，而不只是在操作一个计算机系统。	3.16	1.05	0.70	0.71
D1	PEV	AIGC-supported communicative tasks have helped me learn useful words and expressions in the target language. / AIGC 支持的交际任务帮助我学到目标语中有用的词汇和表达。	3.32	1.11	0.73	0.82
D2	PEV	AIGC-supported communicative tasks have helped me understand how the target language is used in real communication. / AIGC 支持的交际任务帮助我理解目标语在真实交际中的使用方式。	3.96	1.11	0.65	0.55
D3	PEV	AIGC-supported communicative tasks have given me new insight into how to express meaning in the target language. / AIGC 支持的交际任务使我对如何用目标语表达意义有了新的认识。	3.06	1.04	0.65	0.55
D4	PEV	AIGC-supported communicative tasks have helped me understand why certain expressions are appropriate in certain situations. / AIGC 支持的交际任务使我理解了为什么某些表达在特定情境中是恰当的。	3.41	1.11	0.58	0.69
D5	PEV	AIGC-supported communicative tasks have improved my ability to make sense of expressions I had not encountered before. / AIGC 支持的交际任务提高了我理解以往未曾接触过的表达的能力。	3.29	1.09	0.74	0.78
E1	LE	I enjoy taking part in AIGC-supported communicative tasks. / 我喜欢参与 AIGC 支持的交际任务。	3.36	1.11	0.78	0.87
E2	LE	I have fun when I use AIGC to practice communicating in the target language. / 当我使用 AIGC 练习目标语交际时，我感到有趣。	3.78	1.14	0.75	0.84
E3	LE	I find AIGC-supported communicative tasks interesting. / 我觉得 AIGC 支持的交际任务很有意思。	4.02	1.12	0.65	0.64
E4	LE	I find it exciting to come across new expressions or ideas during AIGC-supported communicative tasks. / 在 AIGC 支持的交际任务中遇到新的表达或想法，让我感到兴奋。	3.18	1.04	0.66	0.70
E5	LE	I enjoy interacting with AIGC when practicing communication in the target language. / 在用目标语练习交际时，我享受与 AIGC 互动的过	3.19	1.07	0.71	0.75

		程。				
E6	LE	Overall, I find AIGC-supported communicative tasks enjoyable. / 总体而言，我觉得 AIGC 支持的交际任务令人愉快。	3.98	1.12	0.63	0.60
F1	AT	It does not bother me when an AI response in the target language is not completely clear. / 当 AI 用目标语给出的回应意思不完全清楚时，我并不会为此感到困扰。	3.67	1.11	0.68	0.72
F2	AT	I feel calm when I am unsure whether my target-language expression is fully correct. / 当我不确定自己的目标语表达是否完全正确时，我仍能保持平静。	3.67	1.12	0.68	0.76
F3	AT	I am comfortable with the fact that some parts of AIGC-supported language learning may be vague or incomplete. / 对于 AIGC 支持的语言学习中有些内容可能模糊或不完整，我能够坦然接受。	3.84	1.15	0.71	0.79
F4	AT	I feel at ease when the AI gives a response that is unexpected or hard to interpret. / 当 AI 给出的回应出乎意料或难以理解时，我也能感到自在。	3.21	1.07	0.73	0.78
F5	AT	Uncertainty about meaning in the target language does not make me anxious during AIGC-supported tasks. / 在 AIGC 支持的任务中，意思上的不确定不会让我感到焦虑。	3.47	1.12	0.75	0.72
F6	AT	I am willing to accept differences between the AI's expressions and the ones I have learned before. / 对于 AI 所用的表达与我以前学过的表达之间的差异，我愿意予以接受。	3.12	1.07	0.71	0.72
F7	AT	I find it acceptable not to know exactly how to phrase something in the target language during a task. / 在任务中，即使我不确切知道如何用目标语来组织表达，我也觉得这是可以接受的。	3.73	1.13	0.78	0.86
F8	AT	I see ambiguity in AIGC-supported communication as a natural part of learning rather than as a problem. / 我把 AIGC 支持的交际中的模糊性看作学习中自然的一部分，而不是问题。	3.00	1.04	0.71	0.71

Note. Item-total r = corrected item-total correlation calculated within the item's intended first-order scale. EFA loading = rotated primary loading from the exploratory factor analysis subsample (n = 390). All ambiguity-tolerance items were coded so that higher scores indicate higher ambiguity tolerance.

4 Appendix D. Reliability and convergent validity of first-order and higher-order constructs

Table D1 provides the detailed reliability and convergent-validity results underlying the simplified reliability table in the main text. CTE was modeled as a higher-order construct measured by four first-order dimensions.

Table D1

Reliability and convergent-validity results

Construct	*Level*	*Items*	*alpha*	*CR*	*AVE*	*Loading range*
Behavioral engagement	First-order CTE dimension	4	.870	.869	.623	.770-.833
Cognitive engagement	First-order CTE dimension	4	.845	.845	.579	.650-.814
Agentic engagement	First-order CTE dimension	4	.802	.798	.496	.669-.725
Social-interactive engagement	First-order CTE dimension	4	.848	.850	.590	.603-.825
Communicative task engagement	Higher-order construct	4 dimensions	.784	.823	.538	.679-.766
Social presence	First-order focal construct	4	.837	.822	.537	.691-.805
Perceived epistemic value	First-order focal construct	5	.856	.841	.517	.557-.811
Learning enjoyment	First-order focal construct	6	.885	.869	.527	.626-.830
Ambiguity tolerance	First-order focal construct	8	.914	.914	.572	.703-.807

Note. For CTE, alpha/CR/AVE in the higher-order row are based on the four first-order dimensions. The agentic-engagement AVE was slightly below .50, but CR exceeded .70 and standardized loadings were acceptable; therefore, the dimension was retained.

5 Appendix E. Preliminary measurement comparability across interaction modes

Because H7 and H8 compare patterns across interaction modes, the measurement model was examined separately in the text-based and richer-interaction groups. These tests establish configural comparability only; metric and scalar invariance should be tested in future work before making stronger cross-mode measurement claims.

Table E1

Preliminary measurement comparability across interaction modes (configural model fit within each group)

Group	*n*	*chi-square*	*df*	*CFI*	*TLI*	*RMSEA*

Text-based only	271	737.80	674	.988	.987	.019
Richer interaction group	509	793.05	674	.989	.988	.019

Note. These configural results indicate that the same broad factor structure was tenable in each group. They do not by themselves establish equality of factor loadings or intercepts across modes.

6 Appendix F. Covariate coefficients in regression models

Table F1 reports the coefficients of categorical covariates included in the regression models. Reference categories were male, beginner self-rated proficiency, and rare AIGC use for communicative tasks.

Table F1

Covariate coefficients in regression models

Model	*Covariate contrast*	*b*	*SE*	*p*
H1: LE equation	Gender: Female vs male	-0.112	0.052	0.032
H1: LE equation	Gender: Prefer not to say vs male	0.090	0.116	0.439
H1: LE equation	Proficiency: Intermediate vs beginner	0.117	0.070	0.095
H1: LE equation	Proficiency: Advanced vs beginner	0.110	0.081	0.176
H1: LE equation	AIGC communicative-use frequency: a few times/month vs rarely	-0.078	0.080	0.330
H1: LE equation	AIGC communicative-use frequency: a few times/week vs rarely	-0.153	0.079	0.052
H1: LE equation	AIGC communicative-use frequency: daily vs rarely	-0.050	0.093	0.587
SP equation	Gender: Female vs male	-0.051	0.055	0.359
SP equation	Gender: Prefer not to say vs male	0.062	0.120	0.606
SP equation	Proficiency: Intermediate vs beginner	0.122	0.072	0.090
SP equation	Proficiency: Advanced vs beginner	0.118	0.089	0.182
SP equation	AIGC communicative-use frequency: a few times/month vs rarely	0.099	0.080	0.216
SP equation	AIGC communicative-use frequency: a few times/week vs rarely	-0.073	0.082	0.374
SP equation	AIGC communicative-use frequency: daily vs rarely	0.123	0.094	0.191
PEV equation	Gender: Female vs male	-0.078	0.049	0.107
PEV equation	Gender: Prefer not to say vs male	-0.033	0.094	0.727
PEV equation	Proficiency: Intermediate vs beginner	0.033	0.060	0.580
PEV equation	Proficiency: Advanced vs beginner	0.067	0.075	0.377
PEV equation	AIGC communicative-use frequency: a few times/month vs rarely	-0.077	0.074	0.295
PEV equation	AIGC communicative-use frequency: a few times/week vs rarely	-0.142	0.072	0.048
PEV equation	AIGC communicative-use frequency: daily vs rarely	-0.124	0.085	0.144
LE equation	Gender: Female vs male	-0.029	0.042	0.497
LE equation	Gender: Prefer not to say vs male	0.101	0.090	0.263
LE equation	Proficiency: Intermediate vs beginner	0.048	0.056	0.389
LE equation	Proficiency: Advanced vs beginner	0.052	0.066	0.435

LE equation	AIGC communicative-use frequency: a few times/month vs rarely	-0.106	0.061	0.084
LE equation	AIGC communicative-use frequency: a few times/week vs rarely	-0.105	0.060	0.082
LE equation	AIGC communicative-use frequency: daily vs rarely	-0.069	0.077	0.373

Note. Coefficients are unstandardized estimates with HC3 standard errors. The focal predictors are reported in the main text.

7 Appendix G. Sensitivity analysis excluding social-interactive engagement

Table G1 reports the key sensitivity-analysis results after removing the social-interactive engagement subscale from the CTE composite.

Table G1

Sensitivity analysis excluding social-interactive engagement

Association	***Estimate***	***95% CI***
Reduced CTE -> social presence	0.555	[.481, .629]
Social presence -> perceived epistemic value	0.332	[.270, .394]
Perceived epistemic value -> learning enjoyment	0.396	[.330, .463]
Index of moderated serial mediation	-0.023	[-.037, -.011]

8 Appendix H. Simple-slopes table

Table H1

Simple slopes of communicative task engagement at levels of ambiguity tolerance

**Simple slope of engagement**	**b**	**SE**	**p**	**95% CI**
Engagement → social presence				
Low AT (−1 SD)	0.795	0.052	< .001	[.69, .90]
Mean AT	0.606	0.038	< .001	[.53, .68]
High AT (+1 SD)	0.418	0.056	< .001	[.31, .53]
Engagement → perceived epistemic value				
Low AT (−1 SD)	0.556	0.061	< .001	[.44, .68]
Mean AT	0.443	0.042	< .001	[.36, .53]
High AT (+1 SD)	0.329	0.051	< .001	[.23, .43]
Engagement → learning enjoyment				
Low AT (−1 SD)	0.453	0.054	< .001	[.35, .56]
Mean AT	0.307	0.041	< .001	[.23, .39]
High AT (+1 SD)	0.162	0.046	< .001	[.07, .25]

Note. Slopes are simple slopes of communicative task engagement; SE from HC3 covariance via the delta method; CIs are 95%. SD of ambiguity tolerance = 0.87.
